# Treatments for COVID-19 and acute respiratory infections are associated with gender and comorbidities in an Italian online survey

**DOI:** 10.1371/journal.pone.0342466

**Published:** 2026-02-17

**Authors:** Marco Leonti, Cristina Mollica, Sara Spadaccini, Laura Casu

**Affiliations:** 1 Department of Biomedical Sciences, University of Cagliari, Cittadella Universitaria, Monserrato, Italy; 2 Department of Statistical Sciences, Sapienza University of Rome, Piazzale Aldo Moro, Rome, Italy; 3 Department of Methods and Models Department for Economy, Territory and Finance, Sapienza University of Rome, Rome, Italy; 4 Department of Life and Environmental Sciences, University of Cagliari, Cittadella Universitaria, Monserrato, Italy; University of Naples Federico II: Universita degli Studi di Napoli Federico II, ITALY

## Abstract

Ministries of health need to know and understand factors affecting medical treatment choices of population subgroups, to tailor official recommendations. This study aimed to identify, quantify and compare treatments used by patients with an acute respiratory infection with and without COVID-19 during the first year of the pandemic by accounting for important factors potentially affecting treatment outcomes. With an online questionnaire, retrospective information on treatments used during events of acute respiratory infections were gathered in Italy. Logistic regression was used to detect significant associations between treatments and a set of variables including socio-demographic data, comorbidities and risk factors. We explored differences in treatments used by subjects who developed symptoms of an acute respiratory infection, with and without COVID-19. Women generally took more treatments than men for both, common acute respiratory infections and COVID-19, although, for the latter condition the gender difference in the average number of treatments was smaller. Painkillers (incl. NSAIDs) followed by antibiotics were the most frequently used drugs by both disease groups while the use of macrolide antibiotics and steroids were typical for the COVID group. Logistic regression models for COVID-19 treatments showed significant positive associations between women and the use of food supplements, depression severity and the use of ibuprofen, as well as between both, age class 50−64 and cardiovascular pre-conditions with macrolide antibiotics. COVID-19 patients were largely following official recommendations issued by the Italian Ministry of Health, using less complementary and alternative medicines when compared to common acute respiratory infections. Particularly, official recommendations suggesting the use of allegedly anti-inflammatory macrolide antibiotics for COVID-19 seem to have been followed for patients with cardiovascular pre-conditions. Considering that macrolide antibiotics augment the risk for cardiovascular death and that cardiovascular diseases are known to be a risk factor for COVID-19 related death, treating COVID-19 patients with macrolide antibiotics was probably not a great idea.

## Introduction

The health emergency caused by the COVID-19 pandemic in 2020 led to immediate restrictive governmental measures and treatment recommendations by ministries of health. The quarantine strategy is as old as written history while an early form of social distancing was first applied in the city of Alghero (Northern Sardinia) after an outbreak of plague in the 16th century [1,2]. Both measures were the main tools for containing the spread of COVID-19 worldwide. However, as this was a new disease and pandemic, the medical staff and community were left with the trial-and-error approach regarding the selection of pharmacological interventions and therapy besides some ministerial recommendations. In the absence of effective therapies and traditional knowledge, treatments largely followed those for comparable viral infections, respiratory diseases, and associated symptomatology. The Italian Ministry of Health [3] suggested the use of paracetamol (acetaminophen) and nonsteroidal anti-inflammatory drugs (NSAIDs) for home treatment of COVID-19 associated symptoms including fever, joint and muscle pain. Use of corticosteroids was recommended for hospitalized patients in need of oxygen supplementation and for home treatments of those patients with a worsening of pulse oximetry parameters, requiring oxygen therapy and whose clinical picture did not improve within 72 hours. The use of antibiotics was only considered in case symptoms persisted for more than 72 hours, with a clinical picture suggesting the presence of a bacterial infection overlap or when bacterial infection was demonstrated by culture examination. The use of hydroxychloroquine was advised against due to lack of evidence-based data. Similarly, due to lack of solid and incontrovertible evidence (i.e., from controlled clinical trials) on the efficacy of vitamin supplements and food supplements (e.g., vitamin D, lactoferrin and quercetin), their use was explicitly discouraged [3]. Moreover, vitamin D overdosing can result in vitamin D intoxication and serious adverse effects including hypercalcaemia, renal failure, and tachycardia, which may require hospitalization [4,5]. However, despite official recommendations, and in accordance with personal experiences, preferences, preexisting health conditions and influence by global marketing, patients in Europe and elsewhere typically make use of home remedies and available over-the-counter drugs, including complementary and alternative medicines (CAM) [6–8].

COVID-19 outpatient treatments, outcomes and hospitalization rates of patients treated with a series of prescription and non-prescription drugs and treatment outcomes of hospitalized COVID-19 patients were the focus of several retrospective observational studies in Italy [9–11]. Additionally, compliance with drug therapy in patients with chronic respiratory diseases during the first lockdown in Italy was assessed [12], and gender differences in the treatment of nursing home inmates during the first peak of the pandemic were analysed [13]. Data on the health care behaviour of the general population with respect to acute respiratory infections (ARIs) during the COVID-19 pandemic have so far not been reported from Italy.

With the aim to fill this gap and to obtain an overview of the overall health seeking behaviour, gender differences and compliance with official recommendations for ARI treatments during the first waves of the COVID-19 pandemic in Italy, we analysed data gathered in the context of the ‘Retrospective Treatment and Outcomes study on COVID-19’ (RTO-COVID-19; [14]). We conducted a logistic regression analysis on the use of the most popular treatments in association with a set of relevant covariates to explore treatment differences between subjects who developed symptoms of ARIs with confirmed or probable COVID-19 and those without. Our study assesses health care behaviours (incl. self-treatment and use of prescription drugs) and aspects related to the lack of appropriate guidance on the use of drugs for COVID-19. We focus on contraindications due to the concomitant presence of pre-existing diseases and risk factors as well as gender differences in health care choices of population subgroups.

## Materials and methods

### Survey

The RTO-COVID-19 survey was a large observational and international online study conceived to explore associations between the incidence and outcome of COVID-19 infections with a wide variety of preventive measures and treatments [14]. The survey, running from October 2020 until the end of February 2021, was accessible through a customized Limesurvey platform (https://www.limesurvey.org/) hosted by the University of Geneva.

The original English questionnaire (S1 File) was translated into Italian, back translated and pre-tested with lay persons before starting the survey. Ethic approval of the Italian survey was received by the Ethical committee of the Ethics Committee of the University Hospital of Cagliari (NP/2020/3877; 30/09/2020). Informed consent was obtained from all participants, or their legal guardians and the study performed in accordance with the Declaration of Helsinki.

The survey was disseminated by emails addressed to university employees, including academic and administrative staff across Italy, but also with posts on social media and professional forums to reach out to the more general public. About 95% of the questionnaires were completed in the two-month period between the end of November 2020 and the end of January 2021. Since the vaccination campaign in Italy started 27.12.2020 [15] it hardly interfered with the survey results. Inclusion criteria were a minimum age of 16 years and the capacity to provide consent. Participants were also allowed to eventually complete the survey for members of the same household, in case members could not fill in the questionnaire or below 16 years of age. Once respondents gave their informed consent, the survey proceeded without restrictions. Participation was anonymous and data were stored and treated confidentially.

The questionnaire was structured into six sections: (i) introduction, eligibility criteria and ethics (ii) socio-demographic information, (iii) preventive measures, (iv) symptoms, severity, and outcomes (v) treatments taken, and (vi) medical history and other relevant information. The questions covered over 200 treatments and potential preventive measures including CAM as well as specific activities or exercises. To facilitate compilation, treatments were arranged into homogeneous categories (e.g., conventional treatments, painkillers, antibiotics, herbal preparations, essential oils, vitamins, food supplements) and presented with drop-down menus, so that respondents could select the specific treatments taken or add them manually in the case they were not listed. Moreover, the questionnaire relied on an elaborate conditional branching logic canalizing the survey itinerary along questions relevant to the responses given. Retrospective clinical information was collected by asking participants to report any episode of respiratory illness experienced after January 1^st^, 2020, together with details on the related diagnostic and symptomatic history. Conditionally on the occurrence of an episode of ARI, respondents were asked to specify the treatments and care practices taken during the first week following the diagnosis or symptom onset.

We distinguished the following case definitions:

a *Acute respiratory tract infection*: a symptomatic ARI episode, without evidence for SARS-CoV-2 (Severe acute respiratory syndrome coronavirus 2) infection.
b *Confirmed COVID-19*: a symptomatic ARI episode associated with a positive PCR-test for SARS-CoV-2 within two days prior to or two weeks following symptom onset.
c *Probable COVID-19:* an ARI episode with symptoms indicative of SARS-CoV-2 infection (fever and loss of smell and/or taste).

Here, subjects with an ARI episode and confirmed or probable COVID-19 are referred to as the ‘COVID group’ and those without COVID-19 as the ‘ARI group’. Preventive measures were not part of this analysis.

### Statistical analysis

A summary of the statistics, such as percentage and mean ±sd values were computed to describe the distribution of categorical and numeric variables across the two disease groups. The bivariate association between the use of the most common treatments and each covariate was preliminarily explored with the Chi-squared or Fisher’s exact test, as appropriate. We focused the statistical analysis on treatments taken by a minimum of 50 (15%) COVID and 200 (8%) ARI patients. The set of covariates here considered as potentially affecting the use of treatments were relevant comorbidities, risk factors and socio-demographic data [16]. These covariates included gender, age class, geographical area of residence, economic issues, overweight, respiratory and cardiovascular issues, other comorbidities (incl. diabetes, liver disease and cancer), depression severity, alcohol and nicotine consumption. To account for possible confounding factors affecting the detection of group differences, we relied on the estimation of a series of logistic regression models having the use of the most common treatments as binary outcomes. The logistic regression approach was adopted as a confirmatory multivariable analysis of the exploratory bivariate association tests which cannot control for confounding factors. Due to the categorical nature of the considered covariates, the preliminary check of multicollinearity was conducted by computing all pairwise Cramér’s V coefficients. A Cramér’s V value > 0.7 was assumed as an indication of a strong association and potential multicollinearity.

No imputation technique for missing data was applied, because no method was deemed suitable or indisputable for our study. Consequently, the sample considered in our study is composed of respondents having complete information for all the variables included in the logistic regression models.

We report results obtained with both approaches but are cautious with the interpretation of conventional bivariate tests of association. A *p*-value < 0.05 was considered statistically significant for all tests. The statistical methods were implemented with the R software [17].

## Results

### Descriptive pattern

**Fig 1:**
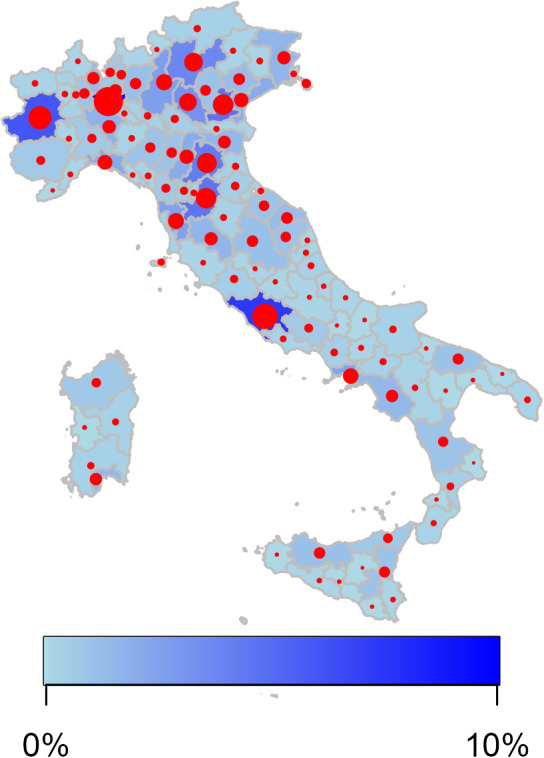
Participation rates in the RTO-COVID-19 survey of respondents by provinces. The map was generated in R by using the ggplot2 package and the Italian geographic boundary data publicly available in the rgeoboundaries package.

From a total of 15567 questionnaires completed (S2 File), 2855 (18.3%) reported contraction of an ARI and were considered for this analysis. These include 2464 (86.3%) reporting the contraction of at least one ARI (without confirmed or probable COVID-19), and 391 (13.7%) reporting confirmed or probable COVID-19. Descriptive summaries of the socio-demographic and clinical characteristics considered in the logistic regression models are provided in Table 1 separately for the two disease groups. The area of Northern and central Italy made a significantly higher contribution to the survey (84%) than the rest of the country (Fig 1). Over 99% respondents were of Caucasian background, and the gender distribution was similar in the two disease groups, with a majority of female participants (59.3% females and 40.7% males in the ARI group and 58.1% females and 41.9% males in the COVID group). During the first week of treatment, patients with simple ARIs used 2.41 treatments on average, whereas patients with confirmed or probable COVID-19 reported a mean of 2.37 treatments.

**Table 1:** Percentage distribution of categorical variables (upper panel) and mean ± sd for numerical variables (lower panel) across disease groups.

Categorical variable	ARI group n = 2464 (86.3%)	COVID group n = 391 (13.7%)
Gender		
Female	59.3	58.1
Male	40.7	41.9
Ethnicity		
Caucasian	99.1	99.4
Other	0.9	0.6
Age class (years)		
16-39	42.6	33.2
40-49	25.1	26.3
50-64	27.4	33.2
≥ 65	4.9	7.2
Geographical area		
North	69.7	77.4
Centre	19.5	15.6
South	10.8	8.0
Economic issues		
No	79.5	78.1
Yes	20.5	21.9
Cardiovascular disease		
No	90.1	87.2
Yes	9.9	12.8
Respiratory disease		
No	94.2	96.4
Yes	5.8	3.6
Other diseases		
No	94.8	94.6
Yes	5.2	5.4
Overweight		
No	67.9	68.8
Yes	32.1	31.2
Nicotine		
No	85.4	94.3
Yes	14.6	5.7
Alcohol Consumption		
Never	17.8	18.4
Once per month	23.0	25.6
2-4 times per month	25.4	26.9
2-3 times per week	20.1	17.2
4+ times per week	13.7	12.0
Depression		
No	63.0	60.0
Mild	23.0	25.0
Moderate	9.0	10.0
Severe	5.0	5.0
**Numerical variable**		
Number of treatments used	2.41 ± 4.2	2.37 ± 3.5

**Fig 2:**
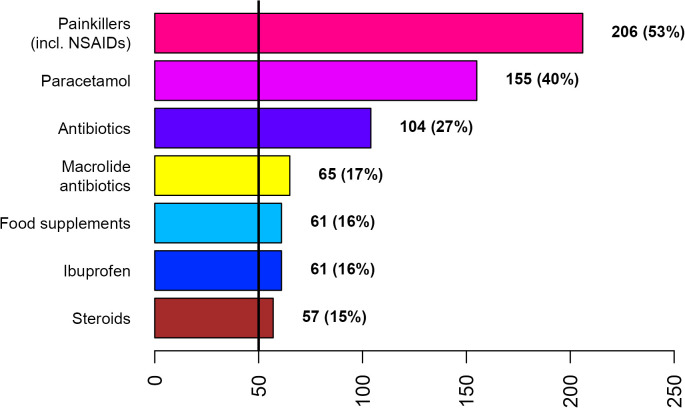
Treatments used by at least 50 COVID patients during the first week after diagnosis or symptom onset.

**Fig 3:**
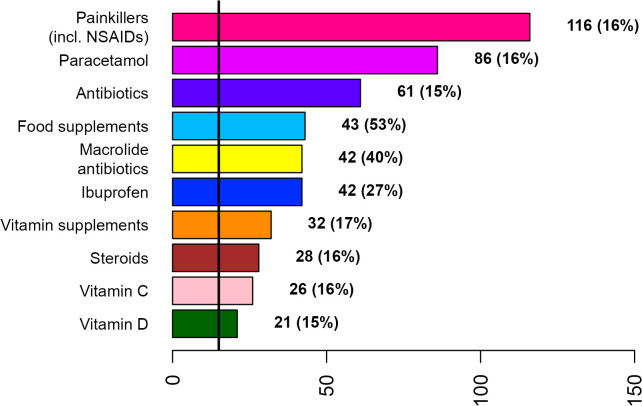
Top 10 treatments used by women for COVID-19 during the first week after diagnosis or symptom onset.

**Fig 4:**
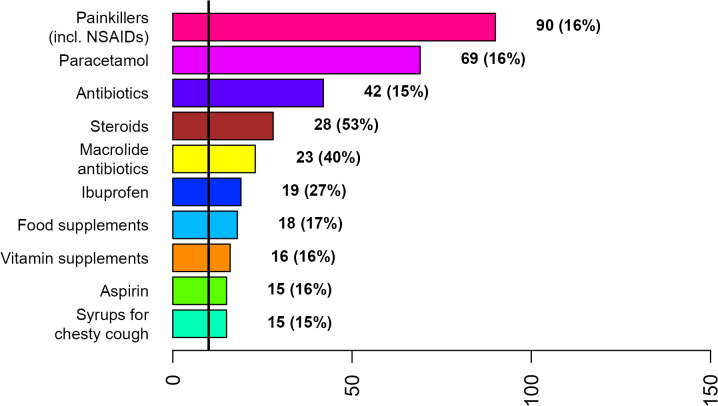
Top 10 treatments used by men for COVID-19 during the first week after diagnosis or symptom onset.

**Fig 5:**
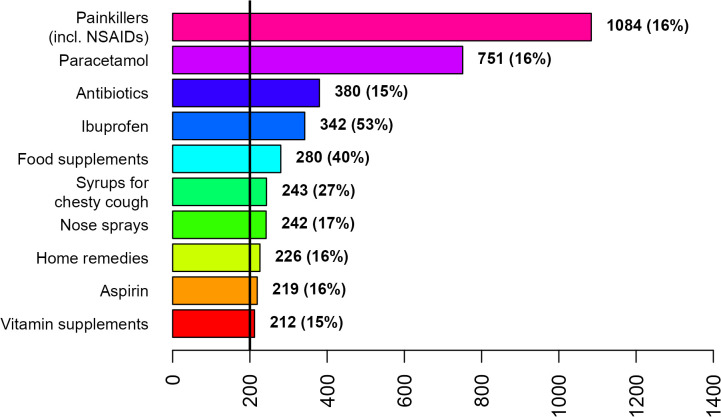
Treatments used by at least 200 ARI patients during the first week after diagnosis or symptom onset.

**Fig 6:**
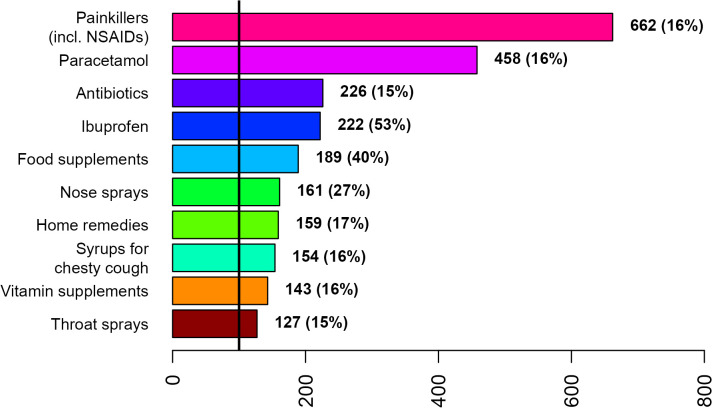
Top 10 treatments used by women for ARIs during the first week after diagnosis or symptom onset.

**Fig 7:**
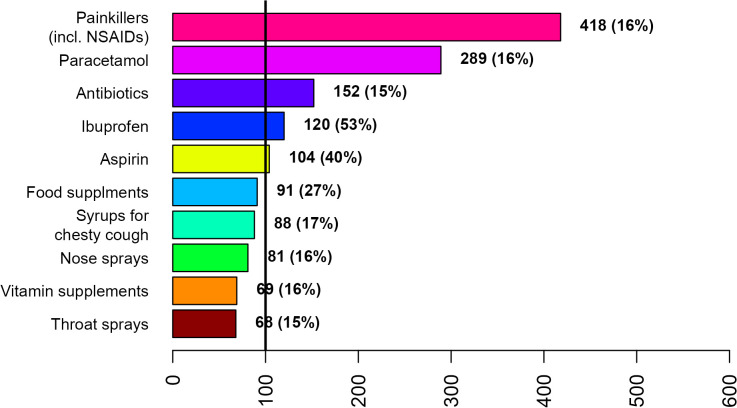
Top 10 treatments used by men for ARIs during the first week after diagnosis or symptom onset.

Painkillers (incl. NSAIDs), paracetamol, and antibiotics were the most frequently used treatments by both disease groups across gender (Figs 2–7; S3 and S4 Tables). Around 53% of the COVID group relied on painkillers and 40% specifically on paracetamol while 46% of the ARI group used painkillers and 31% paracetamol. The use of antibiotics was reported by 26.6% of the COVID group and by 15.4% of the ARI group (Figs 2 and 5). Men generally showed a higher use of aspirin, ketoprofen and naproxen but a lower use of ibuprofen than women in both disease groups (S3 and S4 Tables). Specifically, for treating COVID-19 men also resorted more to paracetamol. The higher reliance on ibuprofen by women and the preference for aspirin by men was more pronounced in the COVID group (Figs 3,4,6 and 7; S3 and S4 Tables).

Other important conventional treatments were sirups for chesty cough (COVID: 7.4%; ARI: 9.9%), nose sprays (COVID: 7.4%; ARI: 9.8%) and steroids (COVID: 14.6%; ARI: 6.2%), with men reporting a higher use of steroids (17.2% males vs. 12.4% females) and treatments for chesty cough (9.2% males vs. 6.2% females) in case of COVID-19. Vitamin and mineral supplementations (COVID: 15.6%; ARI: 11.4%), herbal preparations and home remedies, including nasal rinse and steam inhalation, were the most frequently used CAM treatments (COVID: 6.6%; ARI: 9.2%), and preferentially used by women for either disease group. While the use of vitamin and mineral supplementation was increased in COVID-19 with respect to ARI patients, herbal preparations and home remedies were less frequently used in association with COVID-19. Most frequently reported vitamin supplementations were those with C, D, B12 and multivitamin preparations while magnesium, zinc and calcium were the most frequently reported mineral supplementations for both simple ARI and COVID-19. Preparations derived from ginger (*Zingiber officinale* Roscoe), lemon (*Citrus limon* L.), honey, turmeric (*Curcuma longa* L.) and coneflower (*Echinacea* sp.), and the essential oil of eucalyptus (*Eucalyptus* sp.), tea tree (*Melaleuca alternifolia* (Maiden & Betche) Cheel) and thyme (*Thymus* sp.) applied by steam inhalation were the most frequently used herbal products and home remedies in general (S3 and S4 Tables).

The results of the logistic regression models are presented in Tables 2 and 3 and the associations between covariates and disease groups analysed in the discussion section.

**Table 2:** Results of the logistic regression models for treatments used by at least 50 COVID patients.

Variable	Painkillers (incl. NSAIDs)	Paracetamol	Antibiotics	Macrolide antibiotics	Food suppl.	Ibuprofen	Steroids
**Female**	n.s.	n.s.	n.s.	n.s.	**0.047** **OR 95%CI [1.03,5.64]**	n.s.	n.s.
**Age class: 40–50**	n.s.	n.s.	**0.034** **OR 95%CI [1.08,5.92]**	n.s.	n.s.	n.s.	n.s.
**Age class: 50–64**	n.s.	n.s.	**0.002** **OR 95%CI [1.60,8.10]**	**0.011** **OR 95%CI [1.37,9.82]**	n.s.	n.s.	n.s.
**Age class: ≥ 65**	n.s.	n.s.	n.s.	n.s.	n.s.	n.s.	n.s.
**Geographical area: Centre**	n.s.	n.s.	n.s.	n.s.	n.s.	n.s.	n.s.
**Geographical area: South**	**0.006** **OR 95%CI [0.08,0.64]**	n.s.	n.s.	n.s.	n.s.	n.s.	**0.021** **OR 95%CI [1.16,11.2]**
**Economic issues**	n.s.	n.s.	n.s.	n.s.	n.s.	n.s.	n.s.
**Overweight**	n.s.	n.s.	n.s.	n.s.	**0.028** **OR 95%CI [0.14,0.86]**	n.s.	n.s.
**Respiratory diseases**	n.s.	n.s.	n.s.	n.s.	n.s.	n.s.	n.s.
**Cardiovascular diseases**	n.s.	n.s.	n.s.	**0.045** **OR 95%CI [1.01,7.56]**	n.s.	n.s.	n.s.
**Other comorbidities**	n.s.	n.s.	n.s.	n.s.	n.s.	n.s.	n.s.
**Depression severity: moderate**	n.s.	n.s.	n.s.	n.s.	n.s.	n.s.	n.s.
**Depression severity: severe**	n.s.	n.s.	n.s.	n.s.	n.s.	**0.019** **OR 95%CI [1.28,19.64]**	n.s.
**Alcohol**	n.s.	n.s.	n.s.	n.s.	n.s.	n.s.	n.s.
**Nicotine**	n.s.	n.s.	n.s.	n.s.	n.s.	n.s.	n.s.

Data showing p-values and odds-ratio 95% confidence intervals (OR 95% CI) of significant covariates. n.s. = non-significant values

**Table 3:** Results of the logistic regression models for treatments used by at least 200 ARI patients.

Variable	Painkillers (incl. NSAIDs)	Paracetamol	Antibiotics	Aspirin	Food supplements	Ibuprofen	Syrups chesty cough	Home remedies	Nose Sprays	Vitamin suppl.
**Female**	**0.023** **OR 95%CI [1.03,1.59]**	n.s.	n.s.	n.s.	**0.026** **OR 95%CI [1.05,2.14]**	n.s.	n.s.	**0.010** **OR 95%CI [1.13,2.44]**	n.s.	**0.046** **OR 95%CI [1.01,2.25]**
**Age class 40–49**	n.s.	n.s.	n.s.	n.s.	n.s.	n.s.	n.s.	n.s.	n.s.	
**Age class 50–64**	n.s.	**0.028** **OR 95%CI [0.54,0.97]**	**0.007** **OR 95%CI [1.14,2.31]**	n.s.	**0.007** **OR 95%CI [1.17,2.73]**	n.s.	n.s.	n.s.	n.s.	**0.015** **OR 95%CI [1.12,2.93]**
**Age class ≥65**	n.s.	n.s.	n.s.	n.s.	**0.016** **OR 95%CI [1.15,5.45]**	n.s.	n.s.	n.s.	n.s.	**0.003** **OR 95%CI [1.47,7.33]**
**Geographical area: Centre**	**0.047** **OR 95%CI [0.59,0.99]**	**<0.001** **OR 95%CI [0.45,0.80]**	n.s.	n.s.	n.s.	n.s.	n.s.	n.s.	n.s.	n.s.
**Geographical area: South**	**0.004** **OR 95%CI [0.44,0.85]**	**0.020** **OR 95%CI [0.45,0.93]**	n.s.	**0.041** **OR 95%CI [1.04,4.03]**	n.s.	n.s.	n.s.	n.s.	n.s.	n.s.
**Economic issues**	n.s.	n.s.	n.s.	n.s.	n.s.	n.s.	n.s.	**0.047** **OR 95%CI [1.01,2.25]**	n.s.	**0.006** **OR 95%CI [1.17,2.67]**
**Overweight**	n.s.	n.s.	n.s.	n.s.	n.s.	n.s.	**<0.001** **OR 95%CI [1.29,2.55]**	n.s.	n.s.	n.s.
**Respiratory diseases**	n.s.	n.s.	**<0.001** **OR 95%CI [1.53,3.87]**	n.s.	n.s.	n.s.	n.s.	n.s.	n.s.	n.s.
**Cardiovascular diseases**	**0.032** **OR 95%CI [0.45,0.96]**	n.s.	n.s.	n.s.	n.s.	n.s.	n.s.	n.s.	n.s.	n.s.
**Other comorbidities**	n.s.	**0.006** **OR 95%CI [1.20,3.09]**	**0.049** **OR 95%CI [1.01,2.79]**	n.s.	n.s.	n.s.	n.s.	n.s.	n.s.	n.s.
**Depression severity: mild**	n.s.	n.s.	n.s.	n.s.	n.s.	**0.010** **OR 95%CI [1.10,2.10]**	n.s.	n.s.	n.s.	n.s.
**Depression severity: severe**	n.s.	n.s.	n.s.	n.s.	n.s.	**0.019** **OR 95%CI [1.09,3.26]**	n.s.	n.s.	n.s.	n.s.
**Alcohol: monthly**	n.s.	n.s.	n.s.	n.s.	n.s.	n.s.	**0.001** **OR 95%CI [1.62,5.04]**	n.s.	n.s.	n.s.
**Alcohol: 2–4 times per month**	n.s.	n.s.	n.s.	n.s.	n.s.	n.s.	n.s.	n.s.	n.s.	n.s.
**Alcohol: 2–3 times per week**	n.s.	n.s.	n.s.	**0.013** **OR 95%CI [1.21,4.21]**	**0.012** **OR 95%CI [1.16,3.21]**	n.s.	**0.005** **OR 95%CI [1.30,4.26]**	**0.034** **OR 95%CI [1.06,3.33]**	n.s.	n.s.
**Alcohol: 4+ times per week**	n.s.	n.s.	n.s.	**0.040** **OR 95%CI [1.04,4.03]**	n.s.	n.s.	n.s.	n.s.	n.s.	n.s.
**Nicotine**	n.s.	n.s.	n.s.	n.s.	n.s.	n.s.	n.s.	n.s.	n.s.	n.s.

Data showing p-values and odds-ratio 95% confidence intervals (OR 95% CI) of significant covariates. n.s. = non-significant values.

### Multicollinearity

Concerning the application of the logistic regression models, the preliminary check of multicollinearity showed that all the pairwise Cramer’s V coefficients were well below the critical 0.7 threshold, providing no indication of multicollinearity issues among the covariates (S1 and S2 Fig). The results of the association analysis are discussed in detail in the next section.

## Discussion

### General pattern

It is widely known that symptoms, morbidity, medical care utilization [18] and drug use are gendered [19,20]. In our study, on average women used more treatments for ARI (females: 2.72; males: 1.95) as well as for confirmed or probable COVID-19 (females: 2.53; males: 2.17), which is consistent with the finding that women make generally more use of medications [21,22]. The higher number of treatments taken by women can be interpreted as a higher responsibility for their health status, that might be related to a general lower propensity for engaging in risky behaviours with respect to men [23,24]. However, the fact that women took less treatments for COVID-19 with respect to common ARIs and men took more treatments for COVID-19 with respect to common ARIs suggests that official recommendations were followed and that they had an adjusting effect on treatment behaviours.

### Associations between covariates and COVID-19 treatments

The logistic regression models for treatment of COVID-19 showed age classes 40–49 and 50–64 positively associated with antibiotics in general and age class 50–64 also with macrolide antibiotics in specific. There was also a positive association between patients with cardiovascular preconditions and macrolide antibiotics. While a negative association between overweight patients and the use of dietary supplements was pointed out, women showed a positive association with dietary supplements. Depression severity had a positive association with the use of ibuprofen while patients from the area of southern Italy appeared to have relied less on painkillers but more on steroids with respect to the rest of the country (Table 2). Applying the same model to the ARI group the positive associations between women and food supplements, age class 50–64 and antibiotics, ibuprofen and depression severity as well as the negative association between the use of painkillers (incl. NSAIDs) and southern Italy reemerged (Tables 2 and 3).

The widespread irrational and inappropriate use of antibiotics in southern Europe is well known and associated with multidrug-resistant bacterial stains [25,26]. Macrolides (mycines) were the most relied antibiotics of the COVID group and amoxicillin in combination with clavulanic acid the most frequently used antibiotic in the ARI group by both genders. However, women showed a tendency for using macrolide antibiotics while men for amoxicillin. In an update regarding the use of pharmaceuticals in the therapy of adult patients with COVID-19 released by the Italian Medicines Agency (AIFA) from 05.05.2020 (first published on 09.04.2020), the use of azithromycin was specifically considered [27]. In this communication it was explained that “there is evidence that macrolides exert beneficial effects in patients with inflammatory lung diseases that go beyond the inhibition of replication of pathogenic bacteria”. More specifically, the document reads, that “in vitro and in vivo studies have shown that macrolides mitigate inflammation and modulate the immune system; in particular, they caused downregulation of cell surface adhesion molecules, reduced the production of proinflammatory cytokines, stimulated phagocytosis by alveolar macrophages and inhibited the activation and mobilization of neutrophils”. It is also acknowledged that “the mechanism by which macrolides exert these anti-inflammatory and immunomodulatory effects is not well known” [27]. However, reports about immunomodulatory and anti-inflammatory effects of macrolides in general and azithromycin in specific [28–31] and the hypothesized beneficial effects in case of COVID-19 [27] stand in sharp contrast with the fact that major macrolide antibiotics (azithromycin, clarithromycin and erythromycin) augment the risk for cardiovascular death [32–34] and cardiovascular diseases being a risk factor for COVID-19 related death [15]. The positive association between the use of macrolide antibiotics and COVID patients with pre-existing cardiovascular diseases is thus an issue (Table 2). The generally higher preference of women for using macrolide antibiotics (Fig 3) might be because this class of antibiotics is widely used for treating bacterial infections during pregnancy or because macrolides constitute an alternative to the beta-lactam antibiotics, which were thought to be more allergenic to women [35,36]. A survey among clinicians reported the presence of comorbidities, specific microbial isolates, elevated procalcitonin level, evidence from chest radiography and ultrasound pattern, worsening of patient’s condition, admittance to intensive care unit and orotracheal intubation as the main reasons for antibiotic therapy in COVID-19 patients in Italy [37].

The positive association of ibuprofen with depression severity might point towards depressive states aggravating sensation of malaise or *vice versa*. Cumulative evidence suggests that inflammation and immune mechanisms can cause and contribute to the development of depressive disorders [38,39]. On the other hand, ibuprofen was found to reduce depressive states in patients with osteoarthritis [40]. The higher preference by women for CAM therapies has been highlighted before [20,41–43]. The lower reliance on painkillers (incl. NSAIDs) and higher reliance on steroids in Southern Italy points towards cultural differences across Italy affecting drug use. Nicotine consumption showed no association with any treatment, reflecting the unclear risk potential associated with smoking and COVID-19 noted before [16].

In addition, to the logistic regression analysis the bivariate analysis of the COVID group highlighted a positive association between women and the use of ibuprofen, overweight patients and the use of paracetamol and antibiotics, those having respiratory preconditions with the use of steroids and patients suffering from other comorbidities with the use of antibiotics (S1 Table).

Ibuprofen is the active principle of BuscofenAct indicated as an analgesic in case of pain associated with the menstrual cycle [44] which might explain women’s reliance on this drug. Men might prefer other NSAIDs over ibuprofen because of a perceived association with women’s medicine and the often-pink-coloured confections and tablets. However, although the results of the bivariate analysis have been obtained with standard tests of association, their validity is limited due to the lack of adjustment for confounding factors, which is instead addressed with the estimation of the regression model.

### Associations between covariates and ARI treatments

In the logistic regression model for the treatment of simple ARI, age class 50–64 was negatively associated with the use of paracetamol, but positively with antibiotics, food supplements and vitamins. Women showed positive associations with the use of painkillers (incl. NSAIDs), food supplements, homeopathy and vitamin supplements. ‘Other comorbidities’ were positively associated with paracetamol and antibiotics while age class ≥ 65 showed positive association with food and vitamin supplements (Table 3).

Food supplements are relatively costly over the counter products and might thus be considered less by younger patients with limited economic power. Moreover, similarly to homeopathic treatments, food supplements are generally not backed by evidence-based clinical data, and associated with disease prevention, an aspect neglected by men in general [45,46].

Subjects affected by COPD or asthma are risk groups for viral infections [47]. Patients suffering from pre-existing respiratory diseases (i.e., asthma, chronic obstructive pulmonary disease (COPD), emphysema, chronic bronchitis, pulmonary fibrosis, bronchiectasis) showed significant associations with antibiotics (logistic regression) and with painkillers (incl. NSAIDs) ibuprofen and nose sprays in the bivariate analysis (S2 Table). Obesity is associated with a range of respiratory symptoms and diseases in general [48], a higher risk for contracting respiratory infections [49] and worse outcomes [50]. These relationships might explain the positive association between patients with self-declared overweight and treatments for chesty cough (Table 3) as well as with painkillers (incl. NSAIDs), paracetamol, antibiotics, and nose sprays (bivariate analysis; S2 Table).

The effect of moderate alcohol consumption on symptoms associated with respiratory tract infection is poorly understood [51]. Heavy alcohol consumption impairs cilial clearance [51], but the situation for moderate alcohol consumers is not known. We found no data able to explain increased consumption of aspirin, syrups for chesty cough, food supplements and home remedies by moderate (2–3 times per week) drinkers affected by ARI, unless when assuming that alcohol consumption was not abandoned during illness, which very probably would have exacerbated aftermaths. With or without considering a possible under estimation and whitewashed reporting of patients’ own alcohol consumption, it appears to be associated with higher drug use (**Table 3**).

Subjects with ARI and cardiovascular preconditions showed a negative association with the use of painkillers (incl. NSAIDs) pointing towards patients being aware that treatment with non-aspirin NSAIDs is linked with cardiovascular adverse effects (**Table 3**). In fact, also the use of non-selective cyclooxygenase-2 inhibitors is associated with a higher risk for myocardial infarction, aggravation of heart failure and high blood pressure [52–54]. The positive association between subjects with cardiovascular preconditions and the use of antibiotics found with the COVID group is also present with the ARI group (logistic regression). Higher depression severity was positively associated with the use of ibuprofen (logistic regression), paralleling the results obtained with the COVID group. In addition, the bivariate analysis (S2 Table) found painkillers (incl. NSAIDs), paracetamol, syrups for chesty cough and nose sprays positively associated with depression.

### Limitations of the study

Due to the adopted non-probability sampling, we cannot infer our results on the general Italian population, which is certainly a limitation of this study. We cannot exclude recall bias which could have differently affected responses of the COVID and ARI groups.

## Conclusion

Our study explores treatments for ARI and COVID-19 adopted by a sample of the Italian public during the first wave of the pandemic, based on data gathered from a large-scale observational survey. Although we cannot extrapolate on the general population, our data suggests that Italian patients largely followed official recommendations for COVID-19 treatment. However, besides associations of treatments with comorbidities we found some common treatments significantly associated with gender. Patients relied more on macrolide antibiotics and steroids for COVID-19 and used fewer CAM and home remedies compared to the treatment of common ARI. Women reduced treatments for COVID-19 with respect to common ARIs and men increased treatments for COVID-19 with respect to common ARIs aligning both genders in terms of average number of treatments taken for COVID-19 during the first week. Nevertheless, gender differences concerning the use of paracetamol and NSAIDs were more pronounced in the COVID-19 group. Besides the general adherence to the official guidelines our data also reflects the fact that during the insurgence of new diseases patients tend to choose remedies and medicines for selfcare they are already familiar with. In the specific case these were treatments commonly used for respiratory diseases. The significant positive association between the use of macrolide antibiotics and cardiovascular preconditions in COVID-19 patients seems to have been influenced by official recommendations, but we lack data showing that use of macrolide antibiotics has increased cardiovascular death toll in vulnerable COVID-19 patients.

Our results could inform gender-specific treatment guidelines for future outbreaks of viral pandemics. They point towards the need for a more transparent communication of contraindications of officially recommended treatments and the need for considering regional health care choices in Italy.

Future studies could benefit from incorporating further variables in the analysis, such as on educational level, occupation and body mass index, which were gathered with a low response rate here. By collecting data on symptom severity and integrating a measure for its confounding effect, future studies could also make much needed inferences on treatment outcomes.

## Supporting information

S1 File Survey questionnaire. (PDF)

S2 File Survey responses. (CSV)

S1 Fig Cramér’s V coefficients for the multicollinearity check for the COVID group. (TIF)

S2 Fig Cramér’s V coefficients for the multicollinearity check for the ARI group. (TIF)

S1 Table Tests of association between each covariate and treatments used by at least 50 COVID patients. n.s. = non-significant p-values at level 0.05.
(DOCX)

S2 Table Tests of association between each covariate and treatments used by at least 200 ARI patients. n.s. = non-significant p-values at level 0.05.
(DOCX)

S3 Table Treatments taken for COVID-19. (DOCX)

S4 Table Treatments taken for acute respiratory infections (ARIs). (DOCX)

## References

[1] Quinto Tiberio A. Ectypa pestilentis status Algheriae Sardiniae anni LXXXII et III supra MD. 1584.

[2] Bianucci R, Benedictow OJ, Fornaciari G, Giuffra V. Quinto Tiberio Angelerio and new measures for controlling plague in 16th-century Alghero, Sardinia. Emerg Infect Dis. 2013;19(9):1478–83. doi: 10.3201/eid1909.120311 23968598 PMC3810900

[3] Italian Ministry of Health. doc.: 0024970-30/11/2020-DGPROGS-DGPROGS-P. 2020. https://www.simpios.eu/wp-content/uploads/2020/12/MinistGestioneDomicilio30nov20.pdf

[4] Alkundi A, Momoh R, Musa A, Nwafor N. Vitamin D intoxication and severe hypercalcaemia complicating nutritional supplements misuse. BMJ Case Rep. 2022;15(7):e250553. doi: 10.1136/bcr-2022-250553 35793850 PMC9263930

[5] Maggini V, Crescioli G, Ippoliti I, Gallo E, Menniti-Ippolito F, Chiaravalloti A, et al. Safety profile of Vitamin D in Italy: an analysis of spontaneous reports of adverse reactions related to drugs and food supplements. J Clin Med. 2023;12(14):4726. doi: 10.3390/jcm12144726 37510843 PMC10381134

[6] Fisher P, Ward A. Complementary medicine in Europe. BMJ. 1994;309(6947):107–11. doi: 10.1136/bmj.309.6947.107 8038643 PMC2540528

[7] Singh S, Ernst E. Trick or treatment: The undeniable facts about alternative medicine. WW Norton & Co Inc. 2009.

[8] Leonti M, Casu L. Traditional medicines and globalization: current and future perspectives in ethnopharmacology. Front Pharmacol. 2013;4:92. doi: 10.3389/fphar.2013.00092 23898296 PMC3722488

[9] Cosentino M, Vernocchi V, Martini S, Marino F, Allasino B, Bàlzola MA, et al. Early outpatient treatment of COVID-19: a retrospective analysis of 392 cases in Italy. J Clin Med. 2022;11(20):6138. doi: 10.3390/jcm11206138 36294461 PMC9605012

[10] Fazio S, Bellavite P, Zanolin E, McCullough PA, Pandolfi S, Affuso F. Retrospective study of outcomes and hospitalization rates of patients in italy with a confirmed diagnosis of early COVID-19 and treated at home within 3 days or after 3 days of symptom onset with prescribed and non-prescribed treatments between November 2020 and August 2021. Med Sci Monit. 2021;27:e935379. doi: 10.12659/MSM.935379 34966165 PMC8725339

[11] Guglielmetti L, Aschieri D, Kontsevaya I, Calabrese F, Donisi A, Faggi A, et al. Treatment for COVID-19-a cohort study from Northern Italy. Sci Rep. 2021;11(1):20964. doi: 10.1038/s41598-021-00243-4 34697322 PMC8545945

[12] Principe R, Di Michele L, Sebastiani A, Savi D, Perrone C, Galluccio G, et al. Self-reported compliance with drug therapy during the first SARS-CoV-2 Italian lockdown in patients with respiratory disease. Ann Ist Super Sanita. 2022;58(2):93–9. doi: 10.4415/ANN_22_02_04 35722795

[13] Spini A, Crescioli G, Donnini S, Ziche M, Collini F, Gemmi F, et al. Sex differences in the utilization of drugs for COVID-19 treatment among elderly residents in a sample of Italian nursing homes. Pharmacoepidemiol Drug Saf. 2022;31(5):489–94. doi: 10.1002/pds.5420 35194891 PMC9088595

[14] Francis NA, Becque T, Willcox M, Hay AD, Lown M, Clarke R, et al. Non-pharmaceutical interventions and risk of COVID-19 infection: survey of U.K. public from November 2020 - May 2021. BMC Public Health. 2023;23(1):389. doi: 10.1186/s12889-023-15209-6 36829127 PMC9951136

[15] Italian Ministry of Health. Vaccine day, le prime vaccinazioni in Italia. https://www.salute.gov.it/portale/nuovocoronavirus/dettaglioMaterialiNuovoCoronavirus.jsp?lingua=italiano&id=53&area=nuovoCoronavirus&menu=vuoto. Accessed 2024 June 15.

[16] Williamson EJ, Walker AJ, Bhaskaran K, Bacon S, Bates C, Morton CE, et al. Factors associated with COVID-19-related death using OpenSAFELY. Nature. 2020;584(7821):430–6. doi: 10.1038/s41586-020-2521-4 32640463 PMC7611074

[17] R Core Team. R: A Language and Environment for Statistical Computing. R Foundation for Statistical Computing, Vienna. 2024. https://www.R-project.org/

[18] Green CA, Pope CR. Gender, psychosocial factors and the use of medical services: a longitudinal analysis. Soc Sci Med. 1999;48(10):1363–72. doi: 10.1016/s0277-9536(98)00440-7 10369437

[19] Karlsson Lind L, von Euler M, Korkmaz S, Schenck-Gustafsson K. Sex differences in drugs: the development of a comprehensive knowledge base to improve gender awareness prescribing. Biol Sex Differ. 2017;8(1):32. doi: 10.1186/s13293-017-0155-5 29065918 PMC5655861

[20] Fjær EL, Landet ER, McNamara CL, Eikemo TA. The use of complementary and alternative medicine (CAM) in Europe. BMC Complement Med Ther. 2020;20(1):108. doi: 10.1186/s12906-020-02903-w 32252735 PMC7137515

[21] Loikas D, Wettermark B, von Euler M, Bergman U, Schenck-Gustafsson K. Differences in drug utilisation between men and women: a cross-sectional analysis of all dispensed drugs in Sweden. BMJ Open. 2013;3(5):e002378. doi: 10.1136/bmjopen-2012-002378 23645921 PMC3646185

[22] Orlando V, Mucherino S, Guarino I, Guerriero F, Trama U, Menditto E. Gender differences in medication use: a drug utilization study based on real world data. Int J Environ Res Public Health. 2020;17(11):3926. doi: 10.3390/ijerph17113926 32492925 PMC7312791

[23] Davidson D, Freudenburg W. Gender and environmental risk concerns: a review and analysis of available research. Environ Behav. 1996;28:302–39.

[24] Harris CR, Jenkins M. Gender differences in risk assessment: why do women take fewer risks than men?. Judgm decis mak. 2006;1(1):48–63. doi: 10.1017/s1930297500000346

[25] Goossens H, Ferech M, Vander Stichele R, Elseviers M, ESAC Project Group. Outpatient antibiotic use in Europe and association with resistance: a cross-national database study. Lancet. 2005;365(9459):579–87. doi: 10.1016/S0140-6736(05)17907-0 15708101

[26] Machowska A, Stålsby Lundborg C. Drivers of irrational use of antibiotics in Europe. Int J Environ Res Public Health. 2018;16(1):27. doi: 10.3390/ijerph16010027 30583571 PMC6338985

[27] AIFA. Azitromicina nella terapia dei pazienti adulti con COVID-19. 2020. https://www.aifa.gov.it/documents/20142/1123276/azitromicina_05.05.2020.pdf

[28] Giamarellos-Bourboulis EJ. Macrolides beyond the conventional antimicrobials: a class of potent immunomodulators. Int J Antimicrob Agents. 2008;31(1):12–20. doi: 10.1016/j.ijantimicag.2007.08.001 17935949

[29] Blasi F, Cazzola M, Tarsia P, Cosentini R, Aliberti S, Santus P, et al. Azithromycin and lower respiratory tract infections. Expert Opin Pharmacother. 2005;6(13):2335–51. doi: 10.1517/14656566.6.13.2335 16218893

[30] Zarogoulidis P, Papanas N, Kioumis I, Chatzaki E, Maltezos E, Zarogoulidis K. Macrolides: from in vitro anti-inflammatory and immunomodulatory properties to clinical practice in respiratory diseases. Eur J Clin Pharmacol. 2012;68(5):479–503. doi: 10.1007/s00228-011-1161-x 22105373

[31] Huckle AW, Fairclough LC, Todd I. Prophylactic antibiotic use in COPD and the potential anti-inflammatory activities of antibiotics. Respir Care. 2018;63(5):609–19. doi: 10.4187/respcare.05943 29463692

[32] Koga T, Imaoka H. Azithromycin and the risk of cardiovascular death. N Engl J Med. 2012;367(8):774; author reply 775. doi: 10.1056/NEJMc1207269 22913696

[33] Wong AYS, Root A, Douglas IJ, Chui CSL, Chan EW, Ghebremichael-Weldeselassie Y, et al. Cardiovascular outcomes associated with use of clarithromycin: population based study. BMJ. 2016;352:h6926. doi: 10.1136/bmj.h6926 26768836

[34] Ray WA, Murray KT, Meredith S, Narasimhulu SS, Hall K, Stein CM. Oral erythromycin and the risk of sudden death from cardiac causes. N Engl J Med. 2004;351(11):1089–96. doi: 10.1056/NEJMoa040582 15356306

[35] Bahat Dinur A, Koren G, Matok I, Wiznitzer A, Uziel E, Gorodischer R, et al. Fetal safety of macrolides. Antimicrob Agents Chemother. 2013;57(7):3307–11. doi: 10.1128/AAC.01691-12 23650169 PMC3697347

[36] Patel N, Cojuc-Konigsberg G, Garcia-Guaqueta D, Shah D, Balasubramaniam D, Mahajan A, et al. Effects of Sex and Gender in Beta-Lactam Antibiotic Allergy: A Systematic Review and Meta-analysis. J Allergy Clinical Immunol. 2024;153(2):AB228. doi: 10.1016/j.jaci.2023.11.733 PMC11717607 39491589

[37] Colaneri M, Valsecchi P, Vecchia M, Di Filippo A, Zuccaro V, Seminari E, et al. What prompts clinicians to start antibiotic treatment in COVID-19 patients? An Italian web survey helps us to understand where the doubts lie. J Glob Antimicrob Resist. 2021;26:74–6. doi: 10.1016/j.jgar.2021.05.014 34118478 PMC8187738

[38] Köhler-Forsberg O, N Lydholm C, Hjorthøj C, Nordentoft M, Mors O, Benros ME. Efficacy of anti-inflammatory treatment on major depressive disorder or depressive symptoms: meta-analysis of clinical trials. Acta Psychiatr Scand. 2019;139(5):404–19. doi: 10.1111/acps.13016 30834514

[39] Drevets WC, Wittenberg GM, Bullmore ET, Manji HK. Immune targets for therapeutic development in depression: towards precision medicine. Nat Rev Drug Discov. 2022;21(3):224–44. doi: 10.1038/s41573-021-00368-1 35039676 PMC8763135

[40] Iyengar RL, Gandhi S, Aneja A, Thorpe K, Razzouk L, Greenberg J, et al. NSAIDs are associated with lower depression scores in patients with osteoarthritis. Am J Med. 2013;126(11):1017.e11-8. doi: 10.1016/j.amjmed.2013.02.037 23993259

[41] Kelner M, Wellman B. Health care and consumer choice: medical and alternative therapies. Soc Sci Med. 1997;45(2):203–12. doi: 10.1016/s0277-9536(96)00334-6 9225408

[42] Dello Buono M, Urciuoli O, Marietta P, Padoani W, De Leo D. Alternative medicine in a sample of 655 community-dwelling elderly. J Psychosom Res. 2001;50(3):147–54. doi: 10.1016/s0022-3999(00)00223-3 11316507

[43] Bishop FL, Lewith GT. Who uses CAM? a narrative review of demographic characteristics and health factors associated with CAM use. Evid Based Complement Alternat Med. 2010;7(1):11–28. doi: 10.1093/ecam/nen023 18955327 PMC2816378

[44] Marjoribanks J, Ayeleke RO, Farquhar C, Proctor M. Nonsteroidal anti-inflammatory drugs for dysmenorrhoea. Cochrane Database Syst Rev. 2015;2015(7):CD001751. doi: 10.1002/14651858.CD001751.pub3 26224322 PMC6953236

[45] Deeks A, Lombard C, Michelmore J, Teede H. The effects of gender and age on health related behaviors. BMC Public Health. 2009;9:213. doi: 10.1186/1471-2458-9-213 19563685 PMC2713232

[46] Hiller J, Schatz K, Drexler H. Gender influence on health and risk behavior in primary prevention: a systematic review. Z Gesundh Wiss. 2017;25(4):339–49. doi: 10.1007/s10389-017-0798-z 32215245 PMC7088168

[47] Heikkinen T, Järvinen A. The common cold. Lancet. 2003;361(9351):51–9. doi: 10.1016/S0140-6736(03)12162-9 12517470 PMC7112468

[48] Zammit C, Liddicoat H, Moonsie I, Makker H. Obesity and respiratory diseases. Int J Gen Med. 2010;3:335–43. doi: 10.2147/IJGM.S11926 21116339 PMC2990395

[49] Maccioni L, Weber S, Elgizouli M, Stoehlker A-S, Geist I, Peter H-H, et al. Obesity and risk of respiratory tract infections: results of an infection-diary based cohort study. BMC Public Health. 2018;18(1):271. doi: 10.1186/s12889-018-5172-8 29458350 PMC5819164

[50] Dixon AE, Peters U. The effect of obesity on lung function. Expert Rev Respir Med. 2018;12(9):755–67. doi: 10.1080/17476348.2018.1506331 30056777 PMC6311385

[51] Sisson JH. Alcohol and airways function in health and disease. Alcohol. 2007;41(5):293–307. doi: 10.1016/j.alcohol.2007.06.003 17764883 PMC2081157

[52] Amer M, Bead VR, Bathon J, Blumenthal RS, Edwards DN. Use of nonsteroidal anti-inflammatory drugs in patients with cardiovascular disease: a cautionary tale. Cardiol Rev. 2010;18(4):204–12. doi: 10.1097/CRD.0b013e3181ce1521 20539104

[53] Varga Z, Sabzwari SRA, Vargova V. Cardiovascular risk of nonsteroidal anti-inflammatory drugs: an under-recognized public health issue. Cureus. 2017;9(4):e1144. doi: 10.7759/cureus.1144 28491485 PMC5422108

[54] Schjerning A-M, McGettigan P, Gislason G. Cardiovascular effects and safety of (non-aspirin) NSAIDs. Nat Rev Cardiol. 2020;17(9):574–84. doi: 10.1038/s41569-020-0366-z 32322101

