# Supplementary material for: Treatments for COVID-19 and acute respiratory infections are associated with gender and comorbidities in an Italian online survey
Source: PLoS One. 2026 Feb 17;21(2):e0342466. doi: 10.1371/journal.pone.0342466 (PMC12912575; doi:10.1371/journal.pone.0342466)
Supplement: S1 File — (PDF) [file pone.0342466.s001.pdf]

Welcome to the survey "your COVID experience": Staying well or being ill during the Covid-19 pandemic?

Tell us about your experience during the pandemic! What have you done to prevent yourself from catching Covid-19? Has it worked?

And if you became ill, did you take any treatments? Whether or not you did, whether you took conventional medications or complementary medicine treatments, home remedies, special diet or exercise, we are interested in your experiences. Did they work? We need to learn from everyone's experiences.

Your answers, combined with those from thousands of other people, will help us understand which preventive measures and treatments work - and which don't.

The questions will take 10-20 minutes to complete! Before you start, please find your diary and any medicines you took for recent coughs or fever, as you will need to answer questions about these.

Section A: Survey "your Covid-19 experience": introduction

A team of doctors and health researchers from the University of Southampton and Universities in Switzerland, Sweden, the Netherlands, Germany, China and other countries are researching what, if anything, people have been doing to prevent themselves and others from catching Covid-19, and if they fall ill, what treatments they may have used.

As Covid-19 is new, we need to learn from everyone's experiences. There are many things we need to understand, for example:

- Why do some people develop serious illness while others don't?

- What stops some people getting it?

- Which treatments, if any work - and which don't?

Should I take part?

Everyone's experience is helpful! The more people answer, the more we will learn about the best ways to prevent and treat Covid-19.

We are interested in replies from people from all ages, whether you are at low risk or high risk from Covid-19, and whether or not you have had symptoms of Covid-19. Whatever age you are, whether or not you have any long-standing health problem, please complete the questionnaire.

If you are not in any 'at risk' group but have older -or 'at risk' - relatives or friends, please ask if they are willing to answer this questionnaire. If they are not in a position to complete the survey by themselves, you can do it for them.

You may have lost close relatives or friends because of Covid-19. If you feel able to complete this questionnaire and report what happened to them, we encourage you to do so. Your information could help us to understand why some people deteriorate and so could help to prevent others from dying. We know it is difficult to do this and we thank you sincerely for this contribution to a better understanding of Covid-19.

What will happen if I take part?

The survey will take 10-20 minutes to complete, depending on which sections you complete. Before you start, please find your diary and any medicines which you took for any recent coughs or fevers, as you will need to answer questions about these. If you have a repeat prescription list of medicines that you took, it may be helpful to have the list at hand when completing the survey.

Are there any benefits in my taking part?

Although there are no direct benefits for you, this survey will improve our understanding of which preventive measures may be best at preventing Covid-19, and which treatments may be useful.

Are there any risks involved?

There are no risks in participating in this survey. However if you were very unwell with Covid-19, were admitted to hospital, and/or lost a loved one to Covid-19, then you may find it upsetting to recall your Covid experience. If you don't want to continue, for whatever reason, you can stop the survey at any time. Of course we would like you to answer all the questions as this will give us the best information, but if you don't want to answer a question, you can skip it. Some questions must be answered but these are just ones that will direct you to different bits of the survey for example whether or not you had symptoms of a respiratory infection.

How can I find out about the results?

The results of this survey will be put on our website [add URL] and will lead to scientific publication. Please be advised that results of this survey will not be available for several months, as we need time to process the data. For more information, visit our website [url].

If you would like us to contact you with the results, you have the option to give your e-mail at the end of the survey.

A1. To find out more about the survey, tick the questions that interest

A2. Will the information I provide be safe?

We will not be naming anyone who completes the questionnaire in any of our reports.

Your data will be processed in accordance with the General Data Protection Regulation 2016 (GDPR). Information about how your response data will be processed and stored can be found in our Data Privacy Notice [insert link]. If you would like more information please contact [study e-mail address].

Your anonymised information will be shared with researchers in other universities and research institutions as part of research, to make comparisons between countries.

Please tick this box if you consent to take part in this survey:

I agree ☐

Section B: Participant information

Now answer all the following questions on behalf of this person as if she/he was answering for her/himself.

B1. Are you answering for one of your household members, a close relative (parents, grandparents, aunt, uncle) or a close friend?

Yes ☐

No, I am answering for myself ☐

B2. **Please, tell us who you are completing the survey for:**

- Mother ☐
- Father ☐
- Grandmother ☐
- Grandfather ☐
- Aunt ☐
- Uncle ☐
- Close friend ☐
- Partner ☐
- Child ☐
- Other ☐

Other

Section C: Essential information

C1. **What is your gender?**

- Female ☐
- Male ☐
- Other ☐

Other

C2. **How old are you?**

C3. **Were you pregnant during the pandemic?**

- Yes ☐
- No ☐
- Not applicable ☐

C4. Do you have long-standing health issues which started before the Covid-19 pandemic?

- [ ] No health issues
- [ ] Asthma
- [ ] Chronic obstructive pulmonary disease (COPD, emphysema, chronic bronchitis)
- [ ] Other chronic lung disease (e.g. pulmonary fibrosis, bronchiectasis)
- [ ] Cancer currently on active treatment
- [ ] Cancer (not currently on active treatment)
- [ ] Conditions affecting the heart and blood vessels (e.g. heart attack, angina, irregular heart beat, stroke or mini-stroke)
- [ ] Diabetes
- [ ] High blood pressure
- [ ] Liver disease
- [ ] Other

Other

C5. Do you regularly take any of the following medications?

Please note, it is not known if any of these medicines influence whether you will get Covid-19 or if they are useful or not in treating symptoms of Covid-19.

- [ ] Blood pressure medicines
- [ ] Steroid tablets (prednisone, prednisolone, dexamethasone)
- [ ] Medicines to suppress the immune system (e.g. inflammatory bowel disease, psoriasis, transplantation, etc.)
- [ ] Statins (simvastatin (Simcora®, Zocor®, atorvastatin (Atorva®), etc.)
- [ ] No medicines
- [ ] Other

Other

C6. Which medications for blood pressure?

- A medicine ending in "-pril" (e.g. enalapril, ramipril, lisinopril, captopril, perindopril, etc.) ☐
- A medicine ending in "-sartan" (e.g. losartan, candesartan, valsartan, etc.) ☐
- Water tablets (e.g. furosemide (Lasix®), bendroflumethiazide) ☐
- A medicine ending in "-dipine" (e.g. amlodipine, nifedipine, lercanidipine) ☐
- I am taking (other) medication for blood pressure but I don't know which one ☐
- Other ☐

Other

C7. Which immunosuppressive medication?

- Don't know ☐
- methotrexate ☐
- azathioprine ☐
- cyclosporin ☐
- mycophenolate ☐
- cancer chemotherapy ☐
- specialist treatments for rheumatoid arthritis, psoriasis, inflammatory bowel disease, etc. ☐
- Other ☐

Other

Section D: Testing for Covid-19

D1. Have you had a swab test from your nose or throat to check if you have had Covid-19?

- Yes, my test result was POSITIVE for Covid-19 ☐
- Yes, my test result was NEGATIVE for Covid-19 ☐
- No, I have not had this type of test ☐
- I am not sure if I have been tested ☐

D2. How many days after the start of your symptoms was the swab taken?

- less than 7 days ☐
- 7 to 14 days ☐
- more than 14 days ☐
- I've had no symptoms ☐
- Other ☐

Other

D3. Have you had Covid-19 symptoms that lasted 3 or more days?

- Yes ☐
- No ☐

Section E: Symptoms

Please answer all the following questions about the WORST one of these illnesses.

E1. Since the beginning of the Covid-19 pandemic, have you had symptoms of respiratory infections (E.g. cough, cold, sore throat, earache, headache, fever, flu, taste/smell alternation, shortness of breath) ?

Only answer "yes" if these lasted for 3 days or more.

(Please note, we are not asking about symptoms that are usual for you, such as hay fever and COPD).

- Yes ☐
- No ☐

E2. **Have you had more than one respiratory infection lasting 3 days or more since the beginning of Covid-19 pandemic?**

- Yes, I had more than one ☐
- No, I had only one ☐

E3. **Roughly how many days in total did you have symptoms of respiratory infection since the beginning of March? [or change to start of pandemic in each country]**

E4. **Do you suspect this respiratory illness was caused by Covid-19?**

- Don't know ☐
- Yes, suspected by a health professional ☐
- Yes, suspected by myself ☐
- No ☐
- Other ☐

Other

E5. **Please tick all the symptoms that you experienced:**

- Feeling short of breath ☐
- Chillblains ☐
- Cough ☐
- Coughing up phlegm ☐
- Diarrhea ☐
- Earache ☐
- Sore eyes ☐
- Fever, high temperature ☐
- Headache ☐
- Loss of smell or taste ☐
- New aches and pains in muscles / joints ☐
- Nausea and/or vomiting ☐
- Pains in your chest ☐

Runny or blocked nose ☐

Facial pains, blocked sinus ☐

Skin rashes ☐

Sore throat ☐

Other ☐

Other

E6. **How many days were your normal activities restricted due to this illness?**

E7. **At your worst, how unwell did you feel?**

Not at all unwell 0	1	2	3	4	5	6	7	8	9	The most unwell I have ever been
☐	☐	☐	☐	☐	☐	☐	☐	☐	☐	☐

E8. **How concerned were you by your symptoms?**

Not at all concern ed 0	1	2	3	4	5	6	7	8	9	Extreme ly concern ed 10
☐	☐	☐	☐	☐	☐	☐	☐	☐	☐	☐

E9. **Have you completely recovered from your illness? (All symptoms have resolved?)**

Yes ☐

No ☐

Don't know ☐

Other ☐

Other

E10. **How long did it take for you to recover completely?**

E11. Did your symptoms start more than 1 week ago?

Yes ☐

No ☐

E12. On which date did this illness begin? If you cannot remember the exact date, please estimate it.

Section F: Treatment management of Covid-19 (proven or suspected): introduction

In the following section, please tell us whether you did anything to treat your infection, and if so what, including taking no treatment or using any kind of herbal preparations, special foods or food supplements, self-treatments, special activities, medicines prescribed or recommended by a medical doctor, pharmacist, nurse, herbalist, alternative practitioner or any other health practitioners. This section has two parts: What did you use or do in the first week? What happened after the first week?

We will start now with the treatment(s) you (she/he) used to manage your symptoms in the first week.

Please note that we do not make any judgments or recommendations about the treatments listed in this questionnaire.

F1. Did you take any treatment(s), special foods or supplements, or do any special activities, to help your symptoms within the first week of your illness?

Modern/conventional/chemical medicine (painkillers, cough syrups, antibiotics, chloroquine, etc.) ☐

Other treatments and approaches (e.g.herbal preparations, essential oils, food supplements, exercices, selfmedication, homeopathy, Traditional Chinese Medicine, Ayurvedic medicine, etc.) ☐

No treatment ☐

Other ☐

Other

Section G: What did you do during the first week? Modern/Conventional/Chemical medicine

G1. Please select the type(s) of treatment:

Antihistamine antiallergic (loratadine (Claritine®), cetirizine (Cetallerg®), etc.) ☐

Antibiotics ☐

Corticosteroids (prednisolone (Spiricort®, Lodotra®, etc.), betamethasone (Betnesol®), etc.) ☐

Chloroquine ☐

Hydroxychloroquine ☐

Inhalers (antiasthmatic, bronchodilator) ☐

Nose sprays ☐

Painkillers, anti-inflammatories or cough and cold medicines (paracetamol (Dafalgan®), ibuprofen (Brufen®), aspirin®, Neocitran®, etc.) ☐

Syrups, drops, or lozenges for cough with sputum (acetylcystein (Fluimucil®, etc.) ☐

Syrups, drops, or lozenges for dry couch (codeine (Makatussin®), dextrometorphan (Bexin®, Pulmofor®), etc.) ☐

Throat sprays (Neo-Angin®, Mebucaine®, etc.) ☐

Other ☐

Other

G2. Please select the type of painkillers, anti-inflammatories or cough and cold medicines

Nota bene:

The words followed by ® are brand names, while the words without ® are the international names of the substances, which must be indicated on the packaging.

The names of the proposed drugs follow this pattern: international name of the substance (Brand names®), e.g. paracetamol (Dafalgan®, Panadol®).

paracetamol/acetaminophen (Dafalgan®, Panadol®, Doliprane®, etc.) ☐

ibuprofen (Advil®, Brufen®, Nurofen®, etc.) ☐

aspirin (Aspegic®, Aspirin UPSA®, etc.) ☐

Sinupret® ☐

Bronchipret® ☐

Neocitran® ☐

Other painkillers or anti-inflammatories (diclofenac (Voltarene®), naproxen (Apranax®, Proxen®),ketoprofen, Ketum®, etc.) ☐

Other, please specify ☐

Other, please specify

G3. Please select the type of antibiotics

Nota bene:

The words followed by ® are brand names, while the words without ® are the international names of the substances, which must be indicated on the packaging.

The names of the proposed drugs follow this pattern: international name of the substance (Brand names®), e.g. paracetamol (Dafalgan®, Panadol®).

Similarities between the names of the same class are highlighted in bold, for example -cilins for penicillins.

- -mycins (azithromycine (Zithromax®), clarithromycine (Klacid®), erythromycine (Erythrocyine®)) ☐
- penicillins (Ospen®) ☐
- amoxycillin, amoxycillin-clavulanate (Augmentin®, Co-Amoxicilline) ☐
- flucloxacillin, cloxacillin, oxacillin or dicloxacillin ☐
- -floxacin (ciprofloxacin (Cip Eco®), moxifloxacine (Avalox®)) ☐
- doxycycline (Docycline®) ☐
- cephalosporins (cef- (cefaclor (Ceclor®), cefazolin cefipime, cefadroxil, cefixime, cefuroxime) ☐
- I took antibiotics, but I don't know the name ☐
- Other ☐

Other

G4. Please select the type of inhalers:

Nota bene:

The words followed by ® are brand names, while the words without ® are the international names of the substances, which must be indicated on the packaging.

The names of the proposed drugs follow this pattern: international name of the substance (Brand names®), e.g. paracetamol (Dafalgan®, Panadol®).

- salbutamol (Ventolin®, etc.) (short-acting antiasthmatics) ☐
- salmeterol (Serevent®, etc.) (long-acting antiasthmatics) ☐
- Corticosteroids (fluticasone (Axotide®), ciclesonide (Alvesco®), etc.) ☐
- Combination of antiasthmatics and corticosteroids (Flutiform®, Seretide®, Symbicort®, etc.) ☐
- Bronchodilator (Spiriva®, Atrovent®, Dospir®, etc.) ☐
- I used inhaler but I don't know the name ☐
- Other ☐

Other

G5. Please select the type of nose spray(s):

Nota bene:

The words followed by ® are brand names, while the words without ® are the international names of the substances, which must be indicated on the packaging.

The names of the proposed drugs follow this pattern: international name of the substance (Brand names®), e.g. paracetamol (Dafalgan®, Panadol®).

- Steroid nose spray (e.g. Beconase®, Nasonex®) ☐
- Isotonic water spray (Rhinomer®, Prorhinel®, Serophy®, Triofan®) ☐
- Hypertonic water spray (Triomer® solution hypertonique) ☐
- Nasal decongestant (e.g. Nasivine®, Vibrocil®, Triofan®, etc.) ☐
- Olbas® nasal spray ☐

Olbas® nasal inhaler ☐

ProSens® Protecteur (carragelose) ☐

Triofan allergie® ☐

Vick's First Defence® ☐

Vick's inhaler® ☐

I use nose spray but I don't remember the name ☐

Other ☐

Other

Section H: What did you do during the first week? Other treatments and approaches

H1. What kind of other treatments and approaches did you use?

(alphabetic order)

Nota bene: In case you can't find the products you used, feel free to tick different categories to see which products are suggested. You can then uncheck or leave the question blank.

Please note that a product can be found in different categories. We suggest that you tick it only once, when it best suits your use.

Anthroposophy (Weleda®, Wala®) ☐

Essential oils ☐

Exercices or Activities ☐

Food supplements (vitamins, minerals, amino-acids, omegas, etc.) ☐

Herbal medicine (teas, herbal capsules/syrups/drops (etc.) from Phytotherapy, Traditional Western or Chinese Medicine, Ayurveda, e.g. ginger, elderflower, thyme, etc.)) ☐

Home remedies (e.g. drinks (e.g. ginger and lemon), inhalation, nasal rinse, etc.) ☐

Homeopathy ☐

Special foods and diets (e.g. dairy-free, probiotics, honey, fruits, soups, spices, etc.) ☐

No treatment ☐

Other ☐

Other

H2. What is the name of the anthroposophy product(s) you used?

- Don't know ☐
- Apis/Belladonna cum Mercurio (Wala) ☐
- Bronchi plantago (Wala) ☐
- Cinnabar /Pyrit tablets (Weleda) ☐
- Erysidoron (Weleda) ☐
- Cough Elixir (Weleda) ☐
- Nose balm (Wala) ☐
- Nose oil (Wala) ☐
- Plantago bronchial balm (Wala) ☐
- Pertudoron (Cuprum aceticum comp.) (Weleda) ☐
- Petasites comp. (Wala) ☐
- Pneumodoron 1 and 2 (Weleda) ☐
- Tartarus stibiatus (Weleda) ☐
- Other ☐

Other

H3. What kind of exercices or activities have you done to improve your symptoms?

- Sport activities ☐
- Massages, reflexology ☐
- Meditation, Mindfullness ☐
- Physiotherapy ☐
- Sun bath ☐

SPA (sauna, hammam) ☐

Tai chi ☐

Yoga ☐

Walking, hiking ☐

Other ☐

Other

H4. What is the name of the homeopathic remedy(ies) you used?

Don't know ☐

Aconitum ☐

Allium cepa ☐

Antimonium tartaricum ☐

Arnica montana ☐

Belladonna ☐

Bryonia alba ☐

Calcarea carbonica ☐

Causticum ☐

Coccus cacti ☐

Euphrasia officinalis ☐

Drosera ☐

Gelsemium sempervirens ☐

Hepar sulfuris ☐

Ipecacuana ☐

Kalium bichromicum ☐

Lycopodium clavatum ☐

Mercurius solubilis ☐

Oscillococcinum® ☐

Phosphorus ☐

Pulsatilla ☐

Rhus toxicondendron ☐

Spongia ☐

Sulphur ☐

Euphrasia Officinalis ☐

Other ☐

Other

H5. What is the name of the essential oil(s) you used?

Don't know ☐

Eucalyptus ☐

Lemon ☐

Marjoram ☐

Oregano ☐

Ravintsara ☐

Savory ☐

Tea Tree ☐

Thyme ☐

Gelodurat® [Add common mixture] ☐

Other, please specify ☐

Other, please specify

H6. What kind of Thyme?

- Thyme linalool ☐
- Thyme thymol ☐
- Don't know ☐
- Other ☐

Other

H7. What kind of Eucalptus?

- Eucalyptus globulus ☐
- Eucalyptus radiata ☐
- Don't know ☐
- Other ☐

Other

H8. How did you use the essential oil(s)?

- Inhalation ☐
- On the skin ☐
- Swallowed ☐
- Other ☐

Other

H9. What type of herbal medicine did you use?

- Don't know ☐
- Traditional Western medicine, phytotherapy ☐
- Traditional Chinese medicine ☐

Ayurveda ☐

Other ☐

Other

Section I: What did you do during the first week? Medicinal plants, phytotherapy (other than Chinese or Indian)

I1. What type of herbal preparations did you use?

- Don't know ☐
- Herbal capsules, pills, tablets ☐
- Herbal ointments or lotion for external application ☐
- Herbal syrups or drops ☐
- Herbal teas ☐
- Nose sprays ☐

I2. What herbal tea(s) have you been drinking?

- don't know ☐
- Artemisia annua ☐
- Barberry ☐
- Elderflower ☐
- Ginger ☐
- Hibiscus, karkade, bissap ☐
- Lime tree ☐
- Marshmallow ☐
- Horehound (Marrubium vulgare) ☐
- Sage ☐
- Thyme ☐
- Turmeric ☐
- Combination of herbs ☐
- dont' know the name of herbs but know the name of the product ☐

Other ☐

Other

I3. **Please select the combination of herbs you used**

- Don't know ☐
- Sidroga® [Tisane bucco-pharynge] ☐
- Sidroga® [Tisane contre refroidissement] ☐
- Sidroga® [Tisane toux irritative] ☐
- I combined herbs myself ☐
- I used another combination ☐
- Other ☐

Other

I4. **Please specify the brand name of the combination or the name of herbs combined:**

Herb 1

Herb 2

Herb 3

Herb 4

Herb 5

Herb 6

I5. **What is the name of the herbal drops or syrup(s) you used?**

- don't know ☐
- Barberry ☐
- Echinacea ☐
- Elderflower, elderberry ☐

Ginger ☐

Ginseng ☐

Kaloba®, Umckaloabo® ☐

Onion ☐

Plantago syrup (Lindol®) ☐

Sinupret® ☐

Rhodiola rosea ☐

Other ☐

Other

I6. What is the name of the herbal capsule(s), pill(s) or tablet(s) you used? It may be a brand name or the name of the herb(s).

don't know ☐

Angocin® Zeller ☐

Echinacea ☐

Barberry ☐

Bronchipret® ☐

Elderflower or elderberry ☐

Garlic ☐

Gelodurat® ☐

Ginger ☐

Ginseng ☐

Kaloba®, Umckaloabo® ☐

Lemon ☐

Mint ☐

Sinupret® ☐

Turmeric ☐

Vitango® (Rhodiola rosea) ☐

Other ☐

Other

I7. What is the name of the herbal ointment(s)/lotion(s) you used?

If it is a homemade preparation from essential oils, please go to the Essential Oils section.

- Don't know ☐
- St. John's wort oil ☐
- Olbas® ointment ☐
- Pulmex®, Vick VapoRub®, Dampo, etc. [camphor, eucalyptol preparations] ☐
- Tiger balm ☐
- Arnica oil ☐
- Other ☐

Other

I8. What is the name of the nose spray(s) you used

- Prosens® Protecteur (carragelose from Algae) ☐
- Olbas® inhaler ☐
- Olbas® spray ☐
- Vick's First Defence® ☐
- Vick's inhaler® ☐
- Other ☐

Other

Section J: What did you do during the first week? Traditional Chinese Medicine

J1. What did you use in TCM?

- Acupuncture ☐
- Moxibustion ☐
- TCM herbal preparations ☐
- Other ☐

Other

J2. What is the name of the TCM herbal preparations you used?

- Don't know ☐
- Individualized treatment ☐
- Huo-xiang-zheng-qi capsule ☐
- Lian-hua-qing-wen Capsule ☐
- Ma-xin-gan-shi-tang ☐
- Pei-pa-kao cough syrup ☐
- Qing-fei-pai-du decoction ☐
- Re-du-ning injection ☐
- Shen-qi-fu-zheng Injection ☐
- Shuang-huang-lian ☐
- Shu-feng-jie-du capsule ☐
- Tan-re-qing injection ☐
- Tou-jie-qu-wen granules ☐
- Xue-bi-jing injection ☐
- Yin-hua-ping-gan granule ☐
- Yin-qiao-san powder ☐
- Yu-ping-feng-san ☐

Other ☐

Other

Section K: What did you do during the first week? Ayurveda

K1. What is the name of the Ayurvedic remedy(ies) you used?

- Don't know ☐
- AYUSH-64 ☐
- Adathodai Manapagu ☐
- Agastya Rasayanam ☐
- Anuthaila ☐
- Gudduchi tablet / Samsamana Vati ☐
- Kaba sura kudineer ☐
- Sudarsana ghana vati ☐
- Vishasura kudineer ☐
- Other ☐

Other

Section L: What did you do during the first week? Food supplements

L1. Have you taken a product containing only one substance? If yes, please select from the following categories.

- Vitamin supplements ☐
- Mineral supplements ☐
- Amino acid, protein supplements ☐
- Enzyme supplements ☐
- Omega-3 fatty acids (e.g. cod liver oil, linseed oil, algal oils) ☐

Don't know ☐

Other ☐

Other

L2. What is the name of the vitamin(s) you took?

Vitamin A ☐

Vitamin B12 ☐

Vitamin C ☐

Vitamin D ☐

Vitamin E ☐

Don't know ☐

Other, please specify ☐

Other, please specify

L3. What is the name of the mineral supplement(s) you used?

Calcium ☐

Chrome ☐

Copper ☐

Magnesium ☐

Selenium ☐

Zinc ☐

Don't know ☐

Other ☐

Other

L4. **Did you take product(s) combining different supplements (e.g., multivitamins)?**

Don't know

Comment

Yes and the name of the product was:

Comment

Yes but I don't remember its name

Comment

No

Comment

Other

Other

Section M: What did you do during the first week? Special foods and diets

M1. **What kind of special foods and/or diets have you used to help your symptoms?**

Please do not indicate what you usually do, but what you have implemented for your symptoms.

- Special diets (gluten-free, dairy-free, etc.) ☐
- Bee products (honey, propolis, etc.) ☐
- Fruits and vegetables ☐
- Kombucha, kefir ☐
- Probiotics (e.g. Bioflorin®, Lactibiam®) ☐

Seaweed ☐

Soups ☐

Spices ☐

Other ☐

Other

M2. What kind of special diets did you use to help your symptoms?

Please do not indicate what you usually do, but what you have implemented for your symptoms.

Dairy-free ☐

FODMAP ☐

Gluten-free ☐

Low carb diet ☐

Paleo ☐

Vegetarian ☐

Vegan ☐

Other ☐

Other

M3. What kind of bee products did you use?

Honey ☐

Pollen ☐

Propolis ☐

Royal jelly ☐

Other ☐

Other

M4. What fruits and vegetables have you eaten to improve your symptoms?

Please do not indicate what you usually do, but what you have implemented for your symptoms.

- Orange ☐
- Lemon ☐
- Onion ☐
- Garlic ☐
- Carrots ☐
- Other ☐

Other

M5. What type of soups did you use?

Please do not indicate what you usually do, but what you have implemented for your symptoms.

- Onion soup ☐
- Nettle soup ☐
- Spinach soup ☐
- Onion and garlic soup ☐
- Chicken or beef broth ☐
- Vegetable broth ☐

Other ☐

Other

M6. **What spice(s) did you use to help your symptoms?**

- Cloves ☐
- Cardamom ☐
- Ginger ☐
- Hot pepper ☐
- Oregano ☐
- Turmeric ☐
- Other ☐

Other

Section N: What did you do during the first week? Home remedies

N1. **What kind of home remedies have you prepared to treat your symptoms?**

- Apple cider vinegar ☐
- Ginger and honey tea ☐
- Garlic and onion soup ☐
- Onion syrup ☐
- Inhalation (steam) ☐
- Inhalation with essential oils ☐
- Nasal rinse ☐
- Turnip syrup ☐
- Lemon and honey ☐
- Lemon juice ☐

Other ☐

Other

N2. Which essential oils did you use for inhalation?

- Don't know ☐
- Eucalyptus ☐
- Lemon ☐
- Marjoram ☐
- Oregano ☐
- Ravintsara ☐
- Savory ☐
- Tea Tree ☐
- Thyme ☐
- Nasobol® ☐
- Other ☐

Other

N3. What kind of Thyme?

- Thyme linalool ☐
- Thyme thymol ☐
- Don't know ☐
- Other ☐

Other

N4. What kind of Eucalptus?

- Eucalyptus globulus ☐
- Eucalyptus radiata ☐
- Don't know ☐
- Other ☐

Other

Section O: What did you do during the first week? Prescription, advice

O1. Who prescribed/advised you to take this/these first treatment(s)?

- Don't know ☐
- Doctor or nurse ☐
- Family or friends ☐
- Internet ☐
- Pharmacist ☐
- Practitioner of complementary and alternative medicine ☐
- Yourself ☐
- Other ☐

Other

O2. Approximately, after how many days of illness did you consult a general practitioner?

O3. After how many days of symptoms did you start using these treatments?

Section P: How did your symptoms change after the first week?

Please accept our sincere condolences for your loss

P1. After the first week, how did your symptoms change?

- Resolved ☐
- Improved ☐
- No improvement ☐
- Getting worse or developed new symptoms ☐
- Deceased ☐
- Don't know ☐
- Other ☐

Other

P2. Which symptoms became worse?

- Breathing difficulties ☐
- Fever ☐
- Cough ☐
- Sore throat ☐
- Runny or blocked nose ☐
- Sinusitis (facial pains, blocked sinuses) ☐
- Nausea and/or vomiting ☐
- Diarrhea ☐
- Loss of small or taste ☐
- Coughing up phlegm ☐
- Ear infection ☐
- Eye infection (conjunctivitis) ☐
- Pains in your chest ☐

Other, please specify (one symptoms only) ☐

Other, please specify (one symptoms only)

P3. Did you spend at least 1 night in hospital?

- Yes ☐
- No ☐
- Don't know ☐
- Other ☐

Other

Section Q: Hospital Admission (1)

Please accept our sincere condolences for your loss

Q1. How many nights did you spend in hospital?

☐☐

Q2. During your hospitalization, were you:

	Yes	Uncertain	No
admitted to an Intensive Care Unit (ICU) or Intensive Therapy Unit (ITU) during your hospital admission?	☐	☐	☐
given oxygen by a face mask or tubes that went into your nose?	☐	☐	☐
put on a machine that used a face mask to help you breath (without putting a tube down your throat)?	☐	☐	☐
put on a machine to help you breath that involved having a tube put down your throat?	☐	☐	☐
Did you have a tracheostomy (airway tube put into the front of you neck by an operation)?	☐	☐	☐

Q3. **What happened at the end of the hospital admission?**

- Discharged home ☐
- Deceased ☐
- Don't know ☐
- Other ☐

Other

Section R: What did you do after the first week?

R1. **After that first week, whether you used treatment or not, what did you do?**

- Don't know ☐
- Continued the same treatment ☐
- Used another treatment, and stopped the ones mentioned before ☐
- Added a new treatment to all the medicines mentioned before ☐
- Added a new treatment to some of the medicines mentioned before ☐
- No treatment ☐
- Stopped all treatments ☐
- Started a treatment ☐
- Consulted a health professional ☐
- I have only been sick for a week ☐
- Other, please specify ☐

Other, please specify

R2. Where did you consult the health professional about your illness?

- [ ] Online / e-consultation
- [ ] Telephone or video consultation
- [ ] Face-to-face consultation with General Practioners / nurse in the community
- [ ] Face-to-face assessment in hospital / A&E
- [ ] Other, please specify

Other, please specify

R3. What type of teatment(s) did you initiate during this second stage?

- [ ] Modern/conventional/chemical medicine (painkillers, cough syrups, antibiotics, chloroquine, etc.)
- [ ] Other treatments and approaches (e.g.herbal preparations, essential oils, food supplements, exercices, selfmedication, homeopathy, Traditional Chinese Medicine, Ayurvedic medicine, etc.)
- [ ] Other

Other

Section S: What did you do after the first week? Modern/Conventional/Chemical medicine

S1. Please select the type(s) of treatment:

- [ ] Antiallergic (antihistamines: loratadine (Claritine®), cetirizine (Cetallerg®, etc.)
- [ ] Antibiotics
- [ ] Corticosteroids (prednisolone (Spiricort®, Lodotra®, etc.), betamethasone (Betnesol®), etc.)
- [ ] Chloroquine
- [ ] Hydroxychloroquine
- [ ] Inhalers (antiasthmatic, bronchodilator)
- [ ] Nose sprays
- [ ] Painkillers, anti-inflammatories or cough and cold medicines (paracetamol (Dafalgan®), ibuprofen (Brufen®), aspirin®, Neocitran®, etc.)
- [ ] Syrups, drops, or lozenges for cough with sputum (acetylcystein (Fluimucil®, etc.)
- [ ] Syrups, drops, or lozenges for dry couch (codeine (Makatussin®), dextrometorphan (Bexin®, Pulmofor®), etc.)

Throat sprays (Neo-Angin®, Mebucaine®, etc.) ☐

Other ☐

Other

S2. **Please select the type of painkillers, anti-inflammatories or cough and cold medicines**

Nota bene:

The words followed by ® are brand names, while the words without ® are the international names of the substances, which must be indicated on the packaging.

The names of the proposed drugs follow this pattern: international name of the substance (Brand names®), e.g. paracetamol (Dafalgan®, Panadol®).

paracetamol/acetaminophen (Dafalgan®, Panadol®, Doliprane®, etc.) ☐

ibuprofen (Advil®, Brufen®, Nurofen®, etc.) ☐

aspirin (Aspegic®, Aspirin UPSA®, etc.) ☐

Sinupret® ☐

Bronchipret® ☐

Neocitran® ☐

Other painkillers or anti-inflammatories (diclofenac (Voltarene®), naproxen (Apranax®, Proxen®), ketoprofen, Ketum®, etc.) ☐

Other, please specify ☐

Other, please specify

S3. Please select the type of antibiotics

Nota bene:

The words followed by ® are brand names, while the words without ® are the international names of the substances, which must be indicated on the packaging.

The names of the proposed drugs follow this pattern: international name of the substance (Brand names®), e.g. paracetamol (Dafalgan®, Panadol®).

Similarities between the names of the same class are highlighted in bold, for example -cilins for penicillins.

- -mycins (azithromycine (Zithromax®), clarithromycine (Klacid®), erythromycine (Erythrocyine®)) ☐
- penicillins (Ospen®), amoxycillin-clavulanate (Augmentin®, Co-Amoxicilline) ☐
- -floxacin (ciprofloxacin (Cip Eco®), moxifloxacine (Avalox®)) ☐
- doxycycline (Docycline®) ☐
- cephalosporins (cef- (cefaclor (Ceclor®), cefazolin cefipime, cefadroxil, cefixime, cefuroxime) ☐
- I took antibiotics, but I don't know the name ☐
- Other ☐

Other

S4. Please select the type of inhalers:

Nota bene:

The words followed by ® are brand names, while the words without ® are the international names of the substances, which must be indicated on the packaging.

The names of the proposed drugs follow this pattern: international name of the substance (Brand names®), e.g. paracetamol (Dafalgan®, Panadol®).

- salbutamol (Ventolin®, etc.) (short-acting antiasthmatics) ☐
- salmeterol (Serevent®, etc.) (long-acting antiasthmatics) ☐

Corticosteroids (fluticasone (Axotide®), ciclesonide (Alvesco®), etc.) ☐

Combination of antiasthmatics and corticosteroids (Flutiform®, Seretide®, Symbicort®, etc.) ☐

Bronchodilator (Spiriva®, Atrovent®, Dospir®, etc.) ☐

I used inhaler but I don't know the name ☐

Other ☐

Other

S5. **Please select the type of nose spray(s):**

Nota bene:

The words followed by ® are brand names, while the words without ® are the international names of the substances, which must be indicated on the packaging.

The names of the proposed drugs follow this pattern: international name of the substance (Brand names®), e.g. paracetamol (Dafalgan®, Panadol®).

Steroid nose spray (e.g. Beconase®, Nasonex®) ☐

Isotonic water spray (Rhinomer®, Prorhinel®, Serophy®, Triofan®) ☐

Hypertonic water spray (Triomer® solution hypertonique) ☐

Nasal decongestant (e.g. Nasivine®, Vibrocil®, Triofan®, etc.) ☐

Olbas® nasal spray ☐

Olbas® inhaler ☐

ProSens® Protecteur (carragelose) ☐

Triofan allergie® ☐

Vick's First Defence® ☐

Vick's® inhaler ☐

I use nose spray but I don't remember the name ☐

Other

Other

Section T: Who did you do after the first week? Other treatments and approaches

T1. What kind of other treatments and approaches did you use?

(alphabetic order)

Nota bene: In case you can't find the products you used, feel free to tick different categories to see which products are suggested. You can then uncheck or leave the question blank.

Please note that a product can be found in different categories. We suggest that you tick it only once, when it best suits your use.

- Anthroposophy (Weleda®, Wala®) ☐
- Essential oils ☐
- Exercices or Activities ☐
- Food supplements (vitamins, minerals, amino-acids, omegas, etc.) ☐
- Herbal medicine (teas, herbal capsules/syrups/drops (etc.) from Phytotherapy, Traditional Western or Chinese Medicine, Ayurveda, e.g. ginger, elderflower, thyme, etc.) ☐
- Home remedies (e.g. drinks (e.g. ginger and lemon), inhalation, nasal rinse, etc.) ☐
- Homeopathy ☐
- Special foods and diets (e.g. dairy-free, probiotics, honey, fruits, soups, spices, etc.) ☐
- No treatment ☐
- Other ☐

Other

T2. What is the name of the anthroposophy product(s) you used?

- Don't know ☐
- Apis/Belladonna cum Mercurio (Wala) ☐

Bronchi plantago (Wala) ☐

Cinnabar /Pyrit tablets (Weleda) ☐

Erysidoron (Weleda) ☐

Cough Elixir (Weleda) ☐

Nose balm (Wala) ☐

Nose oil (Wala) ☐

Plantago bronchial balm (Wala) ☐

Pertudoron (Cuprum aceticum comp.) (Weleda) ☐

Petasites comp. (Wala) ☐

Pneumodoron 1 and 2 (Weleda) ☐

Tartarus stibiatus (Weleda) ☐

Other ☐

Other

T3. What kind of exercices or activities have you done to improve your symptoms?

Sport activities ☐

Massages, reflexology ☐

Meditation, Mindfullness ☐

Physiotherapy ☐

Sun bath ☐

SPA (sauna, hammam) ☐

Tai chi ☐

Yoga ☐

Walking, hiking ☐

Other ☐

Other

T4. What is the name of the homeopathic remedy(ies) you used?

- Don't know ☐
- Aconitum ☐
- Allium cepa ☐
- Antimonium tartaricum ☐
- Arnica montana ☐
- Belladonna ☐
- Bryonia alba ☐
- Calcarea carbonica ☐
- Causticum ☐
- Coccus cacti ☐
- Euphrasia officinalis ☐
- Drosera ☐
- Gelsemium sempervirens ☐
- Hepar sulfuris ☐
- Ipecacuana ☐
- Kalium bichromicum ☐
- Lycopodium clavatum ☐
- Mercurius solubilis ☐
- Oscillococcinum® ☐
- Phosphorus ☐
- Pulsatilla ☐
- Rhus toxicondendron ☐
- Spongia ☐
- Sulphur ☐

Other ☐

Other

T5. **What is the name of the essential oil(s) you used?**

- Don't know ☐
- Eucalyptus ☐
- Lemon ☐
- Marjoram ☐
- Oregano ☐
- Ravintsara ☐
- Savory ☐
- Tea Tree ☐
- Thyme ☐
- Gelodurat® [Add common mixture] ☐
- Other, please specify ☐

Other, please specify

T6. **How did you use the essential oil(s)?**

- Inhalation ☐
- On the skin ☐
- Swallowed ☐
- Other ☐

Other

T7. **What kind of Thyme?**

- Thyme linalool ☐
- Thyme thymol ☐
- Don't know ☐
- Other ☐

Other

T8. **What kind of Eucalptus?**

- Eucalyptus globulus ☐
- Eucalyptus radiata ☐
- Don't know ☐
- Other ☐

Other

T9. **What type of herbal medicine did you use?**

- Don't know ☐
- Traditional Western medicine, phytotherapy ☐
- Traditional Chinese medicine ☐
- Ayurveda ☐
- Other ☐

Other

Section U: What did you do after the first week? Medicinal plants, phytotherapy (other than chinese or indian)

U1. What type of herbal preparations did you use?

- Don't know ☐
- Herbal capsules, pills, tablets ☐
- Herbal ointments or lotion for external application ☐
- Herbal syrups or drops ☐
- Herbal teas ☐
- Nose sprays ☐

U2. What herbal tea(s) have you been drinking?

- don't know ☐
- Artemisia annua ☐
- Barberry ☐
- Elderflower ☐
- Ginger ☐
- Hibiscus, karkade, bissap ☐
- Lime tree ☐
- Marshmallow ☐
- Horehound (Marrubium vulgare) ☐
- Sage ☐
- Thyme ☐
- Turmeric ☐
- Combination of herbs ☐
- dont' know the name of herbs but know the name of the product ☐
- Other ☐

Other

U3. **Please select the combination of herbs you used**

- Don't know ☐
- Sidroga® [Tisane bucco-pharynge] ☐
- Sidroga® [Tisane contre refroidissement] ☐
- Sidroga® [Tisane toux irritative] ☐
- I combined herbs myself ☐
- I used another combination ☐
- Other ☐

Other

[ ]

U4. **Please specify the brand name of the combination or the name of herbs combined:**

Herb 1	
Herb 2	
Herb 3	
Herb 4	
Herb 5	
Herb 6	

U5. **What is the name of the herbal drops or syrup(s) you used?**

- don't know ☐
- Barberry ☐
- Echinacea ☐
- Elderflower, elderberry ☐
- Ginger ☐
- Ginseng ☐
- Kaloba®, Umckaloabo® ☐
- Onion ☐
- Plantago syrup (Lindol®) ☐

Sinupret®

Rhodiola rosea

Other

Other

U6. What is the name of the herbal capsule(s), pill(s) or tablet(s) you used? It may be a brand name or the name of the herb(s).

don't know

Angocin®

Echinacea

Barberry

Bronchipret®

Elderflower

Garlic

Ginger

Ginseng

Umckaloabo®

Lemon

Mint

Sinupret®

Turmeric

Vitango® [Rhodiola rosea]

Other

Other

U7. What is the name of the herbal ointment(s)/lotion(s) you used?

If it is a homemade preparation from essential oils, please go to the Essential Oils section.

- Don't know ☐
- St. John's wort oil ☐
- Olbas® ointment ☐
- Pulmex®, Vick VapoRub®, etc. [camphor, eucalyptol preparations] ☐
- Tiger balm ☐
- Arnica oil ☐
- Other ☐

Other

U8. What is the name of the nose spray(s) you used

- ProSens® Protecteur (carragelose) ☐
- Olbas® inhaler ☐
- Olbas® spray ☐
- Vick's First Defence® ☐
- Vick's® inhaler ☐
- Other ☐

Other

Section V: What did you after the first week? Traditional Chinese Medicine

V1. What did you use in TCM?

- Acupuncture ☐
- Moxibustion ☐
- TCM herbal preparations ☐

Other ☐

Other

☐

V2. What is the name of the TCM herbal preparations you used?

- Don't know ☐
- Individualized treatment ☐
- Huo-xiang-zheng-qi capsule ☐
- Lian-hua-qing-wen Capsule ☐
- Ma-xin-gan-shi-tang ☐
- Pei-pa-kao cough syrup ☐
- Qing-fei-pai-du decoction ☐
- Re-du-ning injection ☐
- Shen-qi-fu-zheng Injection ☐
- Shuang-huang-lian ☐
- Shu-feng-jie-du capsule ☐
- Tan-re-qing injection ☐
- Tou-jie-qu-wen granules ☐
- Xue-bi-jing injection ☐
- Yin-hua-ping-gan granule ☐
- Yin-qiao-san powder ☐
- Yu-ping-feng-san ☐
- Other ☐

Other

☐

Section W: What did you after the first week? Ayurveda

W1. **What is the name of the Ayurvedic remedy(ies) you used?**

- Don't know ☐
- AYUSH-64 ☐
- Adathodai Manapagu ☐
- Agastya Rasayanam ☐
- Anuthaila ☐
- Gudduchi tablet / Samsamana Vati ☐
- Kaba sura kudineer ☐
- Sudarsana ghana vati ☐
- Vishasura kudineer ☐
- Other ☐

Other

Section X: What did you do after the first week? Food supplements

X1. **Have you taken a product containing only one substance? If yes, please select from the following categories.**

- Vitamin supplements ☐
- Mineral supplements ☐
- Amino acid, protein supplements ☐
- Enzyme supplements ☐
- Omega-3 fatty acids (e.g. cod liver oil, linseed oil, algal oils) ☐
- Don't know ☐

Other ☐

Other

X2. What is the name of the vitamin(s) you took?

- Vitamin A ☐
- Vitamin B12 ☐
- Vitamin C ☐
- Vitamin D ☐
- Vitamin E ☐
- Don't know ☐
- Other, please specify ☐

Other, please specify

X3. What is the name of the mineral supplement(s) you used?

- Calcium ☐
- Chrome ☐
- Copper ☐
- Magnesium ☐
- Selenium ☐
- Zinc ☐
- Don't know ☐
- Other ☐

Other

X4. **Did you take product(s) combining different supplements (e.g., multivitamins)?**

Don't know ☐

Comment

Yes and the name of the product was: ☐

Comment

Yes but I don't remember its name ☐

Comment

No ☐

Comment

Other ☐

Other

Section Y: What did you do after the first week? Special foods and diets

Y1. **What kind of special foods and/or diets have you used to help your symptoms?**

Please do not indicate what you usually do, but what you have implemented for your symptoms.

Special diets (gluten-free, dairy-free, etc.) ☐

Bee products (honey, propolis, etc.) ☐

Fruits and vegetables ☐

Kombucha, kefir ☐

Probiotics (e.g. Bioflorin®, Lactibiam®) ☐

Seaweed ☐

Soups ☐

Spices ☐

Other ☐

Other

Y2. What kind of special diets did you use to help your symptoms?

Please do not indicate what you usually do, but what you have implemented for your symptoms.

Dairy-free ☐

FODMAP ☐

Gluten-free ☐

Low carb diet ☐

Paleo ☐

Vegetarian ☐

Vegan ☐

Other ☐

Other

Y3. What kind of bee products did you use?

Honey ☐

Pollen ☐

Propolis ☐

Royal jelly ☐

Other ☐

Other

Y4. What fruits and vegetables have you eaten to improve your symptoms?

Please do not indicate what you usually do, but what you have implemented for your symptoms.

- Orange ☐
- Lemon ☐
- Onion ☐
- Garlic ☐
- Carrots ☐
- Other ☐

Other

Y5. What type of soups did you use?

- Onion soup ☐
- Nettle soup ☐
- Spinach soup ☐
- Onion and garlic soup ☐
- Chicken or beef broth ☐
- Vegetable broth ☐
- Other ☐

Other

Y6. **What spice(s) did you use to help your symptoms?**

- Cloves ☐
- Cardamom ☐
- Ginger ☐
- Hot pepper ☐
- Oregano ☐
- Turmeric ☐
- Other ☐

Other

Section Z: What did you after the first week? Home remedies

Z1. **What kind of home remedies have you prepared to treat your symptoms?**

- Apple cider vinegar ☐
- Ginger and honey tea ☐
- Garlic and onion soup ☐
- Onion syrup ☐
- Inhalation (steam) ☐
- Inhalation with essential oils ☐
- Nasal rinse ☐
- Turnip syrup ☐
- Lemon and honey ☐
- Lemon juice ☐
- Other ☐

Other

Z2. Which essential oils did you use for inhalation?

- Don't know ☐
- Eucalyptus ☐
- Lemon ☐
- Marjoram ☐
- Oregano ☐
- Ravintsara ☐
- Savory ☐
- Tea Tree ☐
- Thyme ☐
- Nasobol® ☐
- Other ☐

Other

Z3. What kind of Thyme?

- Thyme linalool ☐
- Thyme thymol ☐
- Don't know ☐
- Other ☐

Other

Z4. **What kind of Eucalptus?**

- Eucalyptus globulus ☐
- Eucalyptus radiata ☐
- Don't know ☐
- Other ☐

Other

Section AA: How did your symptoms change after the second week?

Please accept our sincere condolences for your loss

AA1. **How did your symptoms change after this second week?**

- Resolved ☐
- Improved ☐
- No improvement ☐
- Getting worse ☐
- Deceased ☐
- Don't know ☐
- Other ☐

Other

AA2. **After this second week, what did you do?**

- Don't know ☐
- Used another treatment, and stopped the ones mentioned before ☐
- Added a new treatment to all the medicines mentioned before ☐
- Added a new treatment to some of the medicines mentioned before ☐
- No treatment ☐
- Stopped all treatments ☐
- Started a treatment ☐

Consulted a health professional ☐

Continued the same treatment ☐

Other ☐

Other

AA3. **Where did you consult the health professional about your illness?**

Don't know ☐

Online/ e-consultation ☐

Telephone or video consultation ☐

Face-to-face consultation with docotor / nurse in the community ☐

Face-to-face assessment in hospital / A&E ☐

Other ☐

Other

AA4. **Did you spend at least 1 night in hospital after this second week?**

Yes ☐

No ☐

Don't know ☐

Other ☐

Other

Section AB: Hospital Admission (2)

Please accept our sincere condolences for your loss.

AB1. **How many nights did you spend in hospital?**

AB2. During your hospitalization, were you:

	Yes	Uncertain	No
admitted to an Intensive Care Unit (ICU) or Intensive Therapy Unit (ITU) during your hospital admission?	☐	☐	☐
given oxygen by a face mask or tubes that went into your nose?	☐	☐	☐
put on a machine that used a face mask to help you breath (without putting a tube down your throat)?	☐	☐	☐
put on a machine to help you breath that involved having a tube put down your throat?	☐	☐	☐
Did you have a tracheostomy (airway tube put into the front of you neck by an operation)?	☐	☐	☐

AB3. What happened at the end of the hospital admission?

- Discharged home ☐
- Deceased ☐
- Don't know ☐
- Other ☐

Other

Section AC: Change in symptoms

AC1. Have you been left with any ongoing physical symptoms? If yes, which one(s)?

- Chest pain ☐
- Cough ☐
- Headaches ☐
- Muscles aches ☐
- Breathing difficulties ☐
- Problems with concentration or memory (e.g., memory loss) ☐
- Problems with taste or smell (e.g., loss of smell, taste) ☐
- Tiredness ☐
- No ☐

Other

Other

Section AD: Avoiding transmission of Covid-19 (with symptoms)

AD1. Was anyone else in your household ill with symptoms of a respiratory infection for 3 or more days BEFORE you developed your symptoms ?

(E.g. cough, cold, sore throat, earache, headache, fever, flu, taste/smell alternation, shortness of breath)

Yes

No

Don't know

Other

Other

AD2. Think about the time when your household member was ill. To avoid catching the illness, how often did you do:

	Never (or almost)	Sometimes	Quite often	Very often	Always (or almost)	Don't know	Not applicable
wash your hands with soap or alcohol gel before eating?	☐	☐	☐	☐	☐	☐	☐
wash your hands with soap or alcohol gel after you had been close to the person who was unwell?	☐	☐	☐	☐	☐	☐	☐
feel that you were able to maintain social distancing (staying 2m from the person who was ill)?	☐	☐	☐	☐	☐	☐	☐
consciously avoid touching your eyes, mouth or nose?	☐	☐	☐	☐	☐	☐	☐
clean things that might have viruses on them (eg doors, taps, kitchens, bathrooms)?	☐	☐	☐	☐	☐	☐	☐
wear a mask or face covering (such as a scarf over your mouth)?	☐	☐	☐	☐	☐	☐	☐
avoid touching someone else's pets	☐	☐	☐	☐	☐	☐	☐

AD3. Think about the time when you were ill: to prevent other people from catching your virus, how often did you:

	Never (or almost)	Sometimes	Quite often	Very often	Always (or almost)	Don't know	Not applicable
wash your hands with soap or alcohol gel after blowing your nose or coughing / sneezing into your hands?	☐	☐	☐	☐	☐	☐	☐
cover your mouth with crook of your elbow when coughing / sneezing, if you had a cough or sneeze?	☐	☐	☐	☐	☐	☐	☐
feel that you were able to maintain social distancing (staying 2m from other people)?	☐	☐	☐	☐	☐	☐	☐
consciously avoid touching your eyes, mouth or nose?	☐	☐	☐	☐	☐	☐	☐
clean things that might have viruses on them (eg doors, taps, kitchens, bathrooms)?	☐	☐	☐	☐	☐	☐	☐
wear a mask or face covering (such as a scarf over your mouth)?	☐	☐	☐	☐	☐	☐	☐
spend time alone?	☐	☐	☐	☐	☐	☐	☐
stay at home?	☐	☐	☐	☐	☐	☐	☐
avoid touching someone else's pets	☐	☐	☐	☐	☐	☐	☐
use any other approaches to try and prevent spreading your illness to others?	☐	☐	☐	☐	☐	☐	☐

AD4. Were you in complete self-isolation for the duration of your illness, living totally alone and never going out?

- Yes ☐
- No ☐
- Don't know ☐

AD5. In the 2 weeks before you fell ill, how many times a day did you wash your hands with soap or alcohol gel on average?

Please think back throught the day from getting up, having meals, going to the toilet, going out, coming back, etc.

- 0-2 times ☐
- 3-4 times ☐
- 5-6 times ☐
- 7-9 times ☐
- 10 times or more ☐

AD6. Think back to the two weeks BEFORE your worst episode of illness: how often did you:

	Never (or almost)	Sometimes	Quite often	Very often	Always (almost)	Don't know	Not applicable
wash your hands with soap or alcohol gel when you came into a house (eg after work / shopping / travelling)?	☐	☐	☐	☐	☐	☐	☐
wash your hands with soap or gel before eating?	☐	☐	☐	☐	☐	☐	☐
did you feel that you were able to maintain social distancing (staying 2m from others)?	☐	☐	☐	☐	☐	☐	☐
consciously avoid touching your eyes, mouth or nose?	☐	☐	☐	☐	☐	☐	☐
clean things that might have viruses on them (eg doors, taps, kitchens, bathrooms)?	☐	☐	☐	☐	☐	☐	☐
wear a mask or face covering (such as a scarf over your mouth)?	☐	☐	☐	☐	☐	☐	☐
use any other approaches to try and protect yourself from catching Covid-19? (such as diet, taking vitamins, using nasal sprays, taking herbals or other medicines?	☐	☐	☐	☐	☐	☐	☐
avoid touching someone else's pets?	☐	☐	☐	☐	☐	☐	☐

AD7. In the two weeks before your symptoms, how often were you in crowded places for at least 15 minutes (e.g., shops, public transportation, workplace, pubs, restaurants, sporting events, theatre, movies, conferences, meetings)?

	Never	1-2 times	3-4 times	5-6 times	7-9 times	10 or more
10 to 100 people	☐	☐	☐	☐	☐	☐
with more than 100 people	☐	☐	☐	☐	☐	☐

AD8. Was anyone else in your household ill with symptoms of a respiratory infection (lasting 3 or more days) AFTER you developed your symptoms?

- Yes ☐
- No ☐
- Don't know ☐
- Other ☐

Other

☐

AD9. In a typical week before you had symptoms, on how many days did you do a total of 30 minutes or more of physical activity, which was enough to raise your breathing rate?

☐☐☐☐☐☐☐☐☐☐

Section AE: Avoiding transmission of Covid-19 (positive without symptoms)

AE1. Was anyone else in your household ill with symptoms of a respiratory infection for 3 or more days BEFORE you tested positive?

(E.g. cough, cold, sore throat, earache, headache, fever, flu, taste/smell alternation, shortness of breath)

- Yes ☐
- No ☐
- Don't know ☐
- Other ☐

Other

☐

AE2. Think about the time when your household member was ill. To avoid catching the illness, how often did you do:

	Never (or almost)	Sometimes	Quite often	Very often	Always (or almost)	Don't know	Not applicable
wash your hands with soap or alcohol gel before eating?	☐	☐	☐	☐	☐	☐	☐
wash your hands with soap or alcohol gel after you had been close to the person who was unwell?	☐	☐	☐	☐	☐	☐	☐
feel that you were able to maintain social distancing (staying 2m from the person who was ill)?	☐	☐	☐	☐	☐	☐	☐
consciously avoid touching your eyes, mouth or nose?	☐	☐	☐	☐	☐	☐	☐
clean things that might have viruses on them (e.g. doors, taps, kitchens, bathrooms)?	☐	☐	☐	☐	☐	☐	☐
wear a mask or face covering (such as a scarf over your mouth)?	☐	☐	☐	☐	☐	☐	☐
avoid touching someone else's pets	☐	☐	☐	☐	☐	☐	☐

AE3. Think about to the weeks after your positive diagnosis: to prevent other people from catching your virus, how often did you:

	Never (or almost)	Sometimes	Quite often	Very often	Always (or almost)	Don't know	Not applicable
wash your hands with soap or alcohol gel after blowing your nose or coughing / sneezing into your hands?	☐	☐	☐	☐	☐	☐	☐
cover your mouth with crook of your elbow when coughing / sneezing, if you had a cough or sneeze?	☐	☐	☐	☐	☐	☐	☐
feel that you were able to maintain social distancing (staying 2m from other people)?	☐	☐	☐	☐	☐	☐	☐
consciously avoid touching your eyes, mouth or nose?	☐	☐	☐	☐	☐	☐	☐
clean things that might have viruses on them (e.g. doors, taps, kitchens, bathrooms)?	☐	☐	☐	☐	☐	☐	☐
wear a mask or face covering (such as a scarf over your mouth)?	☐	☐	☐	☐	☐	☐	☐
spend time alone?	☐	☐	☐	☐	☐	☐	☐
stay at home?	☐	☐	☐	☐	☐	☐	☐
avoid touching someone else's pets	☐	☐	☐	☐	☐	☐	☐
use any other approaches to try and prevent spreading your illness to others?	☐	☐	☐	☐	☐	☐	☐

AE4. Were you in complete self-isolation during the 2 weeks following your positive test, living totally alone and never going out?

- Yes ☐
- No ☐
- Don't know ☐

AE5. In the 2 weeks before your positive diagnosis, how many times a day did you wash your hands with soap or alcohol gel on average?

Please think back through the day from getting up, having meals, going to the toilet, going out, coming out, etc.

- 0-2 times ☐
- 3-4 times ☐
- 5-6 times ☐
- 7-9 times ☐
- 10 times or more ☐

AE6. Think back to the two weeks BEFORE your test: how often did you:

	Never (or almost)	Sometimes	Quite often	Very often	Always (almost)	Don't know	Not applicable
wash your hands with soap or alcohol gel when you came into a house (eg after work / shopping / travelling)?	☐	☐	☐	☐	☐	☐	☐
wash your hands with soap or alcohol gel before eating?	☐	☐	☐	☐	☐	☐	☐
did you feel that you were able to maintain social distancing (staying 2m from others)?	☐	☐	☐	☐	☐	☐	☐
consciously avoid touching your eyes, mouth or nose?	☐	☐	☐	☐	☐	☐	☐
clean things that might have viruses on them (eg doors, taps, kitchens, bathrooms)?	☐	☐	☐	☐	☐	☐	☐
wear a mask or face covering (such as a scarf over your mouth)?	☐	☐	☐	☐	☐	☐	☐
use any other approaches to try and protect yourself from catching Covid-19? (such as diet, taking vitamins, using nasal sprays, taking herbals or other medicines?	☐	☐	☐	☐	☐	☐	☐
avoid touching someone else's pets?	☐	☐	☐	☐	☐	☐	☐

AE7. In the two weeks before your positive diagnosis, how often were you in crowded places for at least 15 minutes (e.g., shops, public transportation, workplace, pubs, restaurants, sporting events, theatre, movies, conferences, meetings)?

	Never	1-2 times	3-4 times	5-6 times	7-9 times	10 or more
10 to 100 people	☐	☐	☐	☐	☐	☐
with more than 100 people	☐	☐	☐	☐	☐	☐

AE8. Was anyone else in your household ill with symptoms of a respiratory infection (lasting 3 or more days) AFTER you tested positive?

- Yes ☐
- No ☐
- Don't know ☐
- Other ☐

Other

☐

AE9. **In a typical week before your positive diagnosis, on how many days did you do a total of 30 minutes or more of physical activity, which was enough to raise your breathing rate?**

Section AF: What did you do to avoid catching Covid-19? [asymptomatic]

AF1. **Has anyone else in your household been ill with symptoms of a respiratory infection (lasting 3 or more days) since March 2020? [or change to start of epidemic in that country]**

(E.g. cough, cold, sore throat, earache, headache, fever, flu, taste/smell alternation, shortness of breath)

- Yes ☐
- No ☐
- don't know ☐
- Other ☐

Other

AF2. **Think about the time when your household member was ill. What did you do to avoid catching it?**

	Never (or almost)	Sometimes	Quite often	Very often	Always (or almost)	Don't know	Not applicable
wash your hands with soap or alcohol gel when you came into a house (eg after work / shopping / travelling)?	☐	☐	☐	☐	☐	☐	☐
wash your hands with soap or alcohol gel before eating?	☐	☐	☐	☐	☐	☐	☐
wash your hands with soap or gel after you had been close to the person who was unwell?	☐	☐	☐	☐	☐	☐	☐
feel that you were able to maintain social distancing (staying 2m from the person who was ill)?	☐	☐	☐	☐	☐	☐	☐
consciously avoid touching your eyes, mouth or nose?	☐	☐	☐	☐	☐	☐	☐
clean things that might have viruses on them (eg doors, taps, kitchens, bathrooms)?	☐	☐	☐	☐	☐	☐	☐
wear a mask or face covering (such as a scarf over your mouth)?	☐	☐	☐	☐	☐	☐	☐
avoid touching someone else's pets?	☐	☐	☐	☐	☐	☐	☐

AF3. Before one of your household became ill, how often did you do:

	Never (or almost)	Sometimes	Quite often	Very often	Always (or almost)	Don't know
wash your hands with soap or alcohol gel when you came home (e.g. after work / shopping / travelling)?	☐	☐	☐	☐	☐	☐
you wash your hands with soap or alcohol gel before eating?	☐	☐	☐	☐	☐	☐
feel that you were able to maintain social distancing (staying 2m from others outside your houshold)?	☐	☐	☐	☐	☐	☐
consciously avoid touching your eyes, mouth or nose?	☐	☐	☐	☐	☐	☐
clean things that might have viruses on them (e.g. doors, taps, kitchens, bathrooms)?	☐	☐	☐	☐	☐	☐
wear a mask or face covering (such as a scarf over your mouth)?	☐	☐	☐	☐	☐	☐
use any other approaches to try and protect yourself from catching Covid-19? (such as diet, taking vitamins, using nasal sprays, taking herbals or other medicines?	☐	☐	☐	☐	☐	☐
avoid touching someone else's pets?	☐	☐	☐	☐	☐	☐

AF4. After the start of the Covid-19 pandemic (since start of march [or insert date according to country] 2020), but BEFORE the lockdown, how often did you do:

	Never (or almost)	Sometimes	Quite often	Very often	Always (or almost)	Don't know	Not applicable
wash your hands with soap or alcohol gel when you came home (eg after work / shopping / travelling)?	☐	☐	☐	☐	☐	☐	☐
you wash your hands with soap or alcohol gel before eating?	☐	☐	☐	☐	☐	☐	☐
feel that you were able to maintain social distancing (staying 2m from others outside your household)?	☐	☐	☐	☐	☐	☐	☐
consciously avoid touching your eyes, mouth or nose?	☐	☐	☐	☐	☐	☐	☐
clean things that might have viruses on them (eg doors, taps, kitchens, bathrooms)?	☐	☐	☐	☐	☐	☐	☐
wear a mask or face covering (such as a scarf over your mouth)?	☐	☐	☐	☐	☐	☐	☐
use any other approaches to try and protect yourself from catching Covid-19? (such as diet, taking vitamins, using nasal sprays, taking herbals or other medicines?	☐	☐	☐	☐	☐	☐	☐
avoid touching someone else's pets?	☐	☐	☐	☐	☐	☐	☐

AF5. DURING the lockdown, how often did you do:

	Never (or almost)	Sometimes	Quite often	Very often	Always (or almost)	Don't know	Not applicable
wash your hands with soap or alcohol gel when you came home (eg after work / shopping / travelling)?	☐	☐	☐	☐	☐	☐	☐
you wash your hands with soap or alcohol gel before eating?	☐	☐	☐	☐	☐	☐	☐
feel that you were able to maintain social distancing (staying 2m from others outside your household)?	☐	☐	☐	☐	☐	☐	☐
consciously avoid touching your eyes, mouth or nose?	☐	☐	☐	☐	☐	☐	☐
clean things that might have viruses on them (eg doors, taps, kitchens, bathrooms)?	☐	☐	☐	☐	☐	☐	☐
wear a mask or face covering (such as a scarf over your mouth)?	☐	☐	☐	☐	☐	☐	☐
use any other approaches to try and protect yourself from catching Covid-19? (such as diet, taking vitamins, using nasal sprays, taking herbals or other medicines?	☐	☐	☐	☐	☐	☐	☐
avoid touching someone else's pets?	☐	☐	☐	☐	☐	☐	☐

AF6. Since the start of the Covid-19 pandemic [and in the two weeks before the shutdown], how many times per week were you in crowded places for at least 15 minutes (e.g., shops, public transportation, workplace, pubs, restaurants, sporting events, theatre, movies, conferences, meetings)?

	Never	1-2 times	3-4 times	5-6 times	7-9 times	10 times or more
with more than 10 people	☐	☐	☐	☐	☐	☐
with more than 100 people	☐	☐	☐	☐	☐	☐

AF7. In the past week, on how many days did you do a total of 30 minutes or more of physical activity, which was enough to raise your breathing rate?

☐☐☐☐☐☐☐☐☐☐

Section AG: What did you do to avoid catching Covid-19? Preventive treatments

AG1. To avoid catching Covid-19, which treatment(s), special foods or supplements did you take, or which activities did you do?

Modern/conventional/chemical medicine (painkillers, chloroquine, etc.) ☐

Other treatments and approaches (e.g.herbal preparations, essential oils, food supplements, exercices, selfmedication, homeopathy, Traditional Chinese Medicine, Ayurvedic medicine, etc.) ☐

Other

Other

Section AH: Who did you do to avoid catching Covid-19? Modern/Conventional/Chemical medicine

AH1. Please select the type(s) of treatment:

- Antiallergic (antihistamines: loratadine (Claritine®), cetirizine (Cetallerg®, etc.)
- Chloroquine
- Hydroxychloroquine
- Inhalers (antiasthmatic, bronchodilator)
- Nose sprays
- Painkillers, anti-inflammatories or cough and cold medicines (paracetamol (Dafalgan®), ibuprofen (Brufen®), aspirin®, Neocitran®, etc.)
- Syrups, drops, or lozenges for cough with sputum (acetylcystein (Fluimucil®, etc.)
- Syrups, drops, or lozenges for dry couch (codeine (Makatussin®), dextrometorphan (Bexin®, Pulmofor®), etc.)
- Throat sprays (Neo-Angin®, Mebucaine®, etc.)
- Other

Other

AH2. Please select the type of painkillers, anti-inflammatories or cough and cold medicines.

Nota bene:

The words followed by ® are brand names, while the words without ® are the international names of the substances, which must be indicated on the packaging.

The names of the proposed drugs follow this pattern: international name of the substance (Brand names®), e.g. paracetamol (Dafalgan®, Panadol®).

- paracetamol/acetaminophen (Dafalgan®, Panadol®, Doliprane®, etc.)

ibuprofen (Advil®, Brufen®, Nurofen®, etc.) ☐

aspirin (Aspegic®, Aspirin UPSA®, etc.) ☐

Sinupret® ☐

Bronchipret® ☐

Neocitran® ☐

Other painkillers or anti-inflammatories (diclofenac (Voltarene®), naproxen (Apranax®, Proxen®), ketoprofen, Ketum®, etc.) ☐

Other, please specify ☐

Other, please specify

AH3. Please select the type of inhalers:

Nota bene:

The words followed by ® are brand names, while the words without ® are the international names of the substances, which must be indicated on the packaging.

The names of the proposed drugs follow this pattern: international name of the substance (Brand names®), e.g. paracetamol (Dafalgan®, Panadol®).

salbutamol (Ventolin®, etc.) (short-acting antiasthmatics) ☐

salmeterol (Serevent®, etc.) (long-acting antiasthmatics) ☐

Corticosteroids (fluticasone (Axotide®), ciclesonide (Alvesco®), etc.) ☐

Combination of antiasthmatics and corticosteroids (Flutiform®, Seretide®, Symbicort®, etc.) ☐

Bronchodilator (Spiriva®, Atrovent®, Dospir®, etc.) ☐

I used inhaler but I don't know the name ☐

Other ☐

Other

AH4. Please select the type of nose spray(s):

Nota bene:

The words followed by ® are brand names, while the words without ® are the international names of the substances, which must be indicated on the packaging.

The names of the proposed drugs follow this pattern: international name of the substance (Brand names®), e.g. paracetamol (Dafalgan®, Panadol®).

- Steroid nose spray (e.g. Beconase®, Nasonex®) ☐
- Isotonic water spray (Rhinomer®, Prorhinel®, Serophy®, Triofan®) ☐
- Hypertonic water spray (Triomer® solution hypertonique) ☐
- Nasal decongestant (e.g. Nasivine®, Vibrocil®, Triofan®, etc.) ☐
- Olbas® nasal spray ☐
- Olbas® nasal inhaler ☐
- ProSens® Protecteur (carragelose) ☐
- Triofan allergie® ☐
- Vick's First Defence® ☐
- Vick's® inhaler ☐
- I use nose spray but I don't remember the name ☐
- Other ☐

Other

Section AI: Who did you do to avoid catching Covid-19? Other treatments and approaches

AI1. What kind of other treatments and approaches did you use?

(alphabetic order)

Nota bene: In case you can't find the products you used, feel free to tick different categories to see which products are suggested. You can then uncheck.

Please note that a product can be found in different categories. We suggest that you tick it only once, when it best suits your use.

- Anthroposophy (Weleda®, Wala®) ☐
- Essential oils ☐
- Exercices or Activities ☐
- Food supplements (vitamins, minerals, amino-acids, omegas, etc.) ☐
- Herbal medicine (teas, herbal capsules/syrups/drops (etc.) from Phytotherapy, Traditional Western or Chinese Medicine, Ayurveda, e.g. ginger, elderflower, thyme, etc.) ☐
- Home remedies (e.g. drinks (e.g. ginger and lemon), inhalation, nasal rinse, etc.) ☐
- Homeopathy ☐
- Special foods and diets (e.g. dairy-free, probiotics, honey, fruits, soups, spices, etc.) ☐
- Other ☐

Other

[ ]

AI2. What is the name of the anthroposophy product(s) you used?

- Don't know ☐
- Apis/Belladonna cum Mercurio (Wala) ☐
- Bronchi plantago (Wala) ☐
- Cinnabar /Pyrit tablets (Weleda) ☐
- Erysidoron (Weleda) ☐
- Cough Elixir (Weleda) ☐
- Nose balm (Wala) ☐
- Nose oil (Wala) ☐

Plantago bronchial balm (Wala) ☐

Pertudoron (Cuprum aceticum comp.) (Weleda) ☐

Petasites comp. (Wala) ☐

Pneumodoron 1 and 2 (Weleda) ☐

Tartarus stibiatus (Weleda) ☐

Other ☐

Other

AI3. What kind of exercices or activities have you done to improve your symptoms?

Sport activities ☐

Massages, reflexology ☐

Meditation, Mindfullness ☐

Physiotherapy ☐

Sun bath ☐

SPA (sauna, hammam) ☐

Tai chi ☐

Yoga ☐

Walking, hiking ☐

Other ☐

Other

AI4. What is the name of the homeopathic remedy(ies) you used?

Don't know ☐

Aconitum ☐

Allium cepa ☐

Antimonium tartaricum ☐

Arnica montana ☐

Belladonna ☐

Bryonia alba ☐

Calcarea carbonica ☐

Causticum ☐

Coccus cacti ☐

Euphrasia officinalis ☐

Drosera ☐

Gelsemium sempervirens ☐

Hepar sulfuris ☐

Ipecacuana ☐

Kalium bichromicum ☐

Lycopodium clavatum ☐

Mercurius solubilis ☐

Oscillococcinum® ☐

Phosphorus ☐

Pulsatilla ☐

Rhus toxicondendron ☐

Spongia ☐

Sulphur ☐

Other ☐

Other

AI5. What is the name of the essential oil(s) you used?

Don't know ☐

Eucalyptus ☐

Lemon ☐

Marjoram ☐

Oregano ☐

Ravintsara ☐

Savory ☐

Tea Tree ☐

Thyme ☐

Gelodurat® [Add common mixture] ☐

Other, please specify ☐

Other, please specify

AI6. **How did you use the essential oil(s)?**

Inhalation ☐

On the skin ☐

Swallowed ☐

Other ☐

Other

AI7. **What kind of Thyme?**

Thyme linalool ☐

Thyme thymol ☐

Don't know ☐

Other ☐

Other

AI8. What kind of Eucalptus?

- Eucalyptus globulus ☐
- Eucalyptus radiata ☐
- Don't know ☐
- Other ☐

Other

AI9. What type of herbal medicine did you use?

- Don't know ☐
- Traditional Western medicine, phytotherapy ☐
- Traditional Chinese medicine ☐
- Ayurveda ☐
- Other ☐

Other

Section AJ: Who did you do to avoid catching Covid-19? Traditional Western herbal medicine and phytotherapy

AJ1. What type of herbal preparations did you use?

- Don't know ☐
- Herbal capsules, pills, tablets ☐
- Herbal ointments or lotion for external application ☐
- Herbal syrups or drops ☐
- Herbal teas ☐
- Nose sprays ☐

AJ2. What herbal tea(s) have you been drinking?

- don't know ☐
- Artemisia annua ☐

- Barberry ☐
- Elderflower ☐
- Ginger ☐
- Hibiscus, karkade, bissap ☐
- Lime tree ☐
- Marshmallow ☐
- Horehound (Marrubium vulgare) ☐
- Sage ☐
- Turmeric ☐
- Combination of herbs ☐
- dont' know the name of herbs but know the name of the product ☐
- Other ☐

Other

AJ3. Please select the combination of herbs you used

- Don't know ☐
- Sidroga® [Tisane bucco-pharynge] ☐
- Sidroga® [Tisane contre refroidissement] ☐
- Sidroga® [Tisane toux irritative] ☐
- I combined herbs myself ☐
- I used another combination ☐
- Other ☐

Other

AJ4. **Please specify the brand name of the combination or the name of herbs combined:**

Herb 1 ☐

Herb 2 ☐

Herb 3 ☐

Herb 4 ☐

Herb 5 ☐

Herb 6 ☐

AJ5. **What is the name of the herbal drops or syrup(s) you used?**

- don't know ☐
- Barberry ☐
- Echinacea ☐
- Elderflower, elderberry ☐
- Ginger ☐
- Ginseng ☐
- Kaloba®, Umckaloabo® ☐
- Onion ☐
- Plantago syrup (Lindol®) ☐
- Sinupret® ☐
- Rhodiola rosea ☐
- Other ☐

Other

☐

AJ6. **What is the name of the herbal capsule(s), pill(s) or tablet(s) you used? It may be a brand name or the name of the herb(s).**

- don't know ☐
- Angocin® ☐
- Echinacea ☐
- Barberry ☐

Bronchipret® ☐

Elderflower ☐

Garlic ☐

Ginger ☐

Ginseng ☐

Umckaloabo® ☐

Lemon ☐

Mint ☐

Sinupret® ☐

Turmeric ☐

Vitango® [Rhodiola rosea] ☐

Other ☐

Other

AJ7. What is the name of the herbal ointment(s)/lotion(s) you used?

If it is a homemade preparation from essential oils, please go to the Essential Oils section.

Don't know ☐

St. John's wort oil ☐

Olbas® ointment ☐

Pulmex®, Vick VapoRub®, etc. [camphor, eucalyptol preparations] ☐

Tiger balm ☐

Arnica oil ☐

Other ☐

Other

AJ8. What is the name of the nose spray(s) you used

- Prosens® Protecteur (carragelose from Algae) ☐
- Olbas® inhaler ☐
- Olbas® spray ☐
- Vick's First Defence® ☐
- Vick's® inhaler ☐
- Other ☐

Other

Section AK: What did you do to avoid catching Covid-19? Traditional Chinese Medicine

AK1. What did you use in TCM?

- Acupuncture ☐
- Moxibustion ☐
- TCM herbal preparations ☐
- Other ☐

Other

AK2. What is the name of the TCM herbal preparations you used?

- Don't know ☐
- Individualized treatment ☐
- Huo-xiang-zheng-qi capsule ☐
- Lian-hua-qing-wen Capsule ☐
- Ma-xin-gan-shi-tang ☐
- Pei-pa-kao cough syrup ☐
- Qing-fei-pai-du decoction ☐
- Re-du-ning injection ☐

Shen-qi-fu-zheng Injection ☐

Shuang-huang-lian ☐

Shu-feng-jie-du capsule ☐

Tan-re-qing injection ☐

Tou-jie-qu-wen granules ☐

Xue-bi-jing injection ☐

Yin-hua-ping-gan granule ☐

Yin-qiao-san powder ☐

Yu-ping-feng-san ☐

Other ☐

Other

Section AL: What did you do to avoid catching Covid-19? Ayurveda

AL1. What is the name of the Ayurvedic remedy(ies) you used?

Don't know ☐

AYUSH-64 ☐

Adathodai Manapagu ☐

Agastya Rasayanam ☐

Anuthaila ☐

Gudduchi tablet / Samsamana Vati ☐

Kaba sura kudineer ☐

Sudarsana ghana vati ☐

Vishasura kudineer ☐

Other

Other

Section AM: What did you do to avoid catching Covid-19? Food supplements

AM1. Have you taken a product containing only one substance? If yes, please select from the following categories.

- Vitamin supplements ☐
- Mineral supplements ☐
- Amino acid, protein supplements ☐
- Enzyme supplements ☐
- Omega-3 fatty acids (e.g. cod liver oil, linseed oil, algal oils) ☐
- Don't know ☐
- Other ☐

Other

AM2. What is the name of the vitamin(s) you took?

- Vitamin A ☐
- Vitamin B12 ☐
- Vitamin C ☐
- Vitamin D ☐
- Vitamin E ☐
- Don't know ☐

Other, please specify

Other, please specify

AM3. What is the name of the mineral supplement(s) you used?

Calcium

Chrome

Copper

Magnesium

Selenium

Zinc

Don't know

Other

Other

AM4. Did you take product(s) combining different supplements (e.g., multivitamins)?

Don't know

Comment

Yes and the name of the product was:

Comment

Yes but I don't remember its name

Comment

No

Comment

Other

Other

Section AN: Who did you do to avoid catching Covid-19? Special foods and diets

AN1. What kind of special foods and/or diets have you used to help your symptoms?

Please do not indicate what you usually do, but what you have implemented for your symptoms.

- Special diets (gluten-free, dairy-free, etc.)
- Bee products (honey, propolis, etc.)
- Fruits and vegetables
- Kombucha, kefir
- Probiotics (e.g. Bioflorin®, Lactibiam®)
- Seaweed
- Soups
- Spices
- Other

Other

AN2. What kind of special diets did you use to help your symptoms?

Please do not indicate what you usually do, but what you have implemented for your symptoms.

- Dairy-free

FODMAP ☐

Gluten-free ☐

Low carb diet ☐

Paleo ☐

Vegetarian ☐

Vegan ☐

Other ☐

Other

AN3. What kind of bee products did you use?

Honey ☐

Pollen ☐

Propolis ☐

Royal jelly ☐

Other ☐

Other

AN4. What fruits and vegetables have you eaten to improve your symptoms?

Please do not indicate what you usually do, but what you have implemented for your symptoms.

Orange ☐

Lemon ☐

Onion ☐

Garlic ☐

Carrots ☐

Other ☐

Other

AN5. What type of soups did you use?

Onion soup ☐

Nettle soup ☐

Spinach soup ☐

Onion and garlic soup ☐

Chicken or beef broth ☐

Vegetable broth ☐

Other ☐

Other

AN6. What spice(s) did you use to help your symptoms?

Cloves ☐

Cardamom ☐

Ginger ☐

Hot pepper ☐

Oregano ☐

Turmeric ☐

Other ☐

Other

Section AO: What did you do to avoid catching Covid-19? Home remedies

AO1. What kind of home remedies have you prepared to avoid catching Covid-19?

- Apple cider vinegar ☐
- Ginger and honey tea ☐
- Garlic and onion soup ☐
- Onion syrup ☐
- Nasal rinse ☐
- Inhalation (steam) ☐
- Inhalation with essential oil(s) ☐
- Turnip syrup ☐
- Lemon and honey ☐
- Lemon juice ☐
- Other ☐

Other

AO2. Which essential oils did you use for inhalation?

- Don't know ☐
- Eucalyptus ☐
- Lemon ☐
- Marjoram ☐
- Oregano ☐
- Ravintsara ☐
- Savory ☐
- Tea Tree ☐
- Thyme ☐
- Nasobol® ☐

Other ☐

Other

AO3. What kind of Thyme?

Thyme linalool ☐
Thyme thymol ☐
Don't know ☐
Other ☐

Other

AO4. What kind of Eucalptus?

Eucalyptus globulus ☐
Eucalyptus radiata ☐
Don't know ☐
Other ☐

Other

Section AP: General information

In this last section, we would like to know some of your personal characteristics (age, gender, etc.). It will help us to better picture the various experiences related to COVID-19 infection. This information will remain strictly confidential.

AP1. Have you had a blood or fingerprick test to find out if you have had Covid-19 (antibody tests)?

Yes, my test result was POSITIVE for antibodies to Covid-19 ☐
Yes, I had a test and it was NEGATIVE for antibodies to Covid-19 ☐
No, I did not have this type of test ☐
I am not sure if I was tested ☐

AP2. Have you had a flu vaccine in the past year(s)?

- Yes, in 2019 ☐
- Yes, in 2018 ☐
- No ☐
- Don't know ☐
- Other ☐

Other

AP3. What is your "ethnic group"? (main origin of your family)

(We are asking this question because previous research suggests that some ethnic groups may be at greater risk from Covid-19)

- East Asian origin ☐
- European origin ("Caucasian") ☐
- African origin (south of the Sahara) ☐
- Latin America ☐
- Middle-East and North African origin ☐
- South Asian origin ☐
- Mixed ethnicity ☐
- Don't know ☐
- Other ☐

Other

AP4. In which country do you live?

- Afghanistan ☐
- Albania ☐
- Algeria ☐
- Andorra ☐
- Angola ☐
- Antigua and Barbuda ☐
- Argentina ☐
- Armenia ☐
- Australia ☐
- Austria ☐
- Azerbaijan ☐
- Bahamas ☐
- Bahrain ☐
- Bangladesh ☐
- Barbados ☐
- Belarus ☐
- Belgium ☐
- Belize ☐
- Benin ☐
- Bhutan ☐
- Bolivia ☐
- Bosnia and Herzegovina ☐
- Botswana ☐
- Brazil ☐
- Brunei ☐
- Bulgaria ☐
- Burkina Faso ☐
- Burundi ☐
- Côte d'Ivoire ☐
- Cabo Verde ☐
- Cambodia ☐

AP5. Please indicate your postcode (if applicable)

AP6. What was your main type of work during the Covid-19 pandemic?

- Retired ☐
- Unemployed ☐
- Furloughed ☐
- Working from home ☐
- Working outside of home ☐
- Full-time student ☐
- Part at home and part outside ☐
- Other ☐

Other

AP7. If working outside of home, what work do you do?

- Healthcare worker in hospital (e.g., doctor or nurse or health care assistant) ☐
- Healthcare worker in the community (e.g., doctor or nurse or health care assistant) ☐
- Carer ☐
- Working in a shop / supermarket ☐
- Delivery ☐
- Other ☐

Other

AP8. **How often did you wear appropriate personal protective equipment (PPE) when you were close to people with possible infection?**

- Almost never ☐
- Sometimes ☐
- Quite often ☐
- Very often ☐
- Almost always ☐
- Don't know ☐
- Not applicable ☐

AP9. **How many years of full-time education have you had in total?**

☐☐☐☐☐☐☐☐☐☐

AP10. **Do you provide personal care to someone not living in your household, whether you are paid or not?**

(for example helping in the house with cooking, cleaning, bathing, dressing)

- Yes ☐
- No ☐

AP11. **Did you have any problems because of a lack of money during the pandemic?**

- Not at all ☐
- Some problems ☐
- Big problems ☐
- Huge problems ☐

Section AQ: Household

If you wish, you can provide an email contact for other household members who wish to participate at the end of this survey. If they are not able to use email. please help them complete the questionnaire.

AQ1. **Do you live alone or do you live with other people (e.g. family, friend, flatmates, lodgers, retirement, student residences, etc.)?**

- I live alone ☐
- I live with other people (family, flatmates, residences, etc.) ☐

AQ2. How many people live in your household including yourself?

Children (age < 5) ☐☐

Children (5-17) ☐☐

Adults (age 18-64) ☐☐

Older adults (age > 64) ☐☐

AQ3. Has anyone in your household already completed this survey?

Yes ☐

No ☐

AQ4. Please enter your household code:

☐

AQ5. Has anyone else in your household had to leave the home for work during the lockdown [during the pandemic if no lockdown]?

Yes ☐

No ☐

AQ6. What work does she/he do?

Healthcare worker in hospital (e.g., doctor or nurse or health care assistant) ☐

Healthcare worker in the community (e.g., doctor or nurse or health care assistant) ☐

Carer ☐

Working in a shop / supermarket ☐

Delivery ☐

Other ☐

Other

☐

AQ7. Do you have any cats or dogs in your household?

Yes ☐

No ☐

Don't know ☐

Section AR: Health information

AR1. Over the last 2 weeks, how often have you been bothered by the following problems?

	Not at all	Several days	More than half the days	Nearly every day
Feeling nervous, anxious, or on edge	☐	☐	☐	☐
Not being able to stop or control worrying	☐	☐	☐	☐
Feeling down, depressed, or hopeless	☐	☐	☐	☐
Little interest or pleasure in doing things	☐	☐	☐	☐

AR2. Do you consider yourself:

Underweight ☐

Normal weight ☐

Overweight ☐

AR3. Do you know your height and weight in:

Centimeters and kilograms (cm and kg) ☐

Feet/inches and stones/pounds (ft/in and st/lb) ☐

Don't know ☐

AR4. Please indicate your height in centimeters and your weight in kilograms

Height (cm) ☐☐☐

Weight (kg) ☐☐☐

AR5. Please indicate your height in feet/inches and your weight in stones/pounds:

Height (feet) ☐☐☐

Height (inches) ☐☐☐

Weight (stones) ☐☐☐

Weight (lbs) ☐☐☐

AR6. Do you currently smoke tobacco (cigarettes, cigars, pipe, waterpipe), use electronic cigarettes or smoking cessation aids every day?

I don't know ☐

No ☐

I smoke tobacco ☐

I use electronic cigarettes (vaping) ☐

I use smoking cessation aids containing nicotine ☐

Other ☐

Other

[ ]

AR7. On average, how many of the following products do you currently smoke/use daily?

Manufactured cigarettes ☐☐

Hand-rolled cigarettes ☐☐

Cigars, cigarillos, cherrots ☐☐

Pipes full of tobacco ☐☐

Waterpipe ☐☐

Other, please specify: ☐☐

AR8. Which of the following products do you currently use every day?

Electronic cigarette with nicotine ☐

Electronic cigarette without nicotine ☐

Other, please specify: ☐

Other ☐

Other

[ ]

AR9. Which of the following products do you use every day?

nicotine-containing tablet or gum ☐

spray containing nicotine ☐

patch containing nicotine ☐

Other ☐

Other

AR10. If you know approximately how much nicotine you consume per day, please specify it:

AR11. How often did you have a drink containing alcohol in the past year?

- Never ☐
- Monthly or less ☐
- Two to four times a month ☐
- Two to three times per week ☐
- Four or more times in a week ☐
- Other ☐

Other

AR12. How many drinks containing alcohol did you have on a typical day when you were drinking in the past year?

- 1 or 2 drinks ☐
- 3 or 4 ☐
- 5 or 6 ☐
- 7 to 9 ☐
- 10 or more ☐
- Other ☐

Other

AR13. How often did you have six or more drinks on one occasion in the past year?

- Never ☐
- Less than monthly ☐
- Monthly ☐
- Weekly ☐
- Daily or almost daily ☐
- Other ☐

Other

AR14. What do you usually eat?

- Don't know ☐
- I do not follow any special diet ☐
- Dairy-free ☐
- FODMAP ☐
- Gluten-free ☐
- Low carb diet ☐
- Paleo ☐
- Vegetarian ☐
- Vegan ☐
- Other ☐

Other

Section AS: Source of information

AS1. Where did you find out about this survey?

- Direct email ☐

Friends/family ☐

Online searches ☐

Networks (professional or private) ☐

Newspaper ☐

Radio ☐

Social media ☐

Television news ☐

Other ☐

Other

AS2. Please specify the network:

AS3. Please specify

Facebook ☐

Whatsapp ☐

WeChat ☐

Twitter ☐

Other ☐

Other

Section AT: Follow up

AT1. **If you want, you can enter your email address here.**

This will make it possible to send you a summary of the results, and to contact you again for a brief follow-up survey. However this is purely optional. If you do provide your e-mail, it will be stored securely and will not be shared with anyone else.

AT2. **If you want to leave a comment on this questionnaire, please write it here:**

Thank you for sharing your experience with us. We hope you enjoyed this survey.

Please share this survey in your social media network: link…

If you have any questions related to this study, please contact us: email address…

You can also visit our website for more information on the survey: http….
