# Supplementary figures and images for: Treatments for COVID-19 and acute respiratory infections are associated with gender and comorbidities in an Italian online survey
Source: PLoS One. 2026 Feb 17;21(2):e0342466. doi: 10.1371/journal.pone.0342466 (PMC12912575; doi:10.1371/journal.pone.0342466)

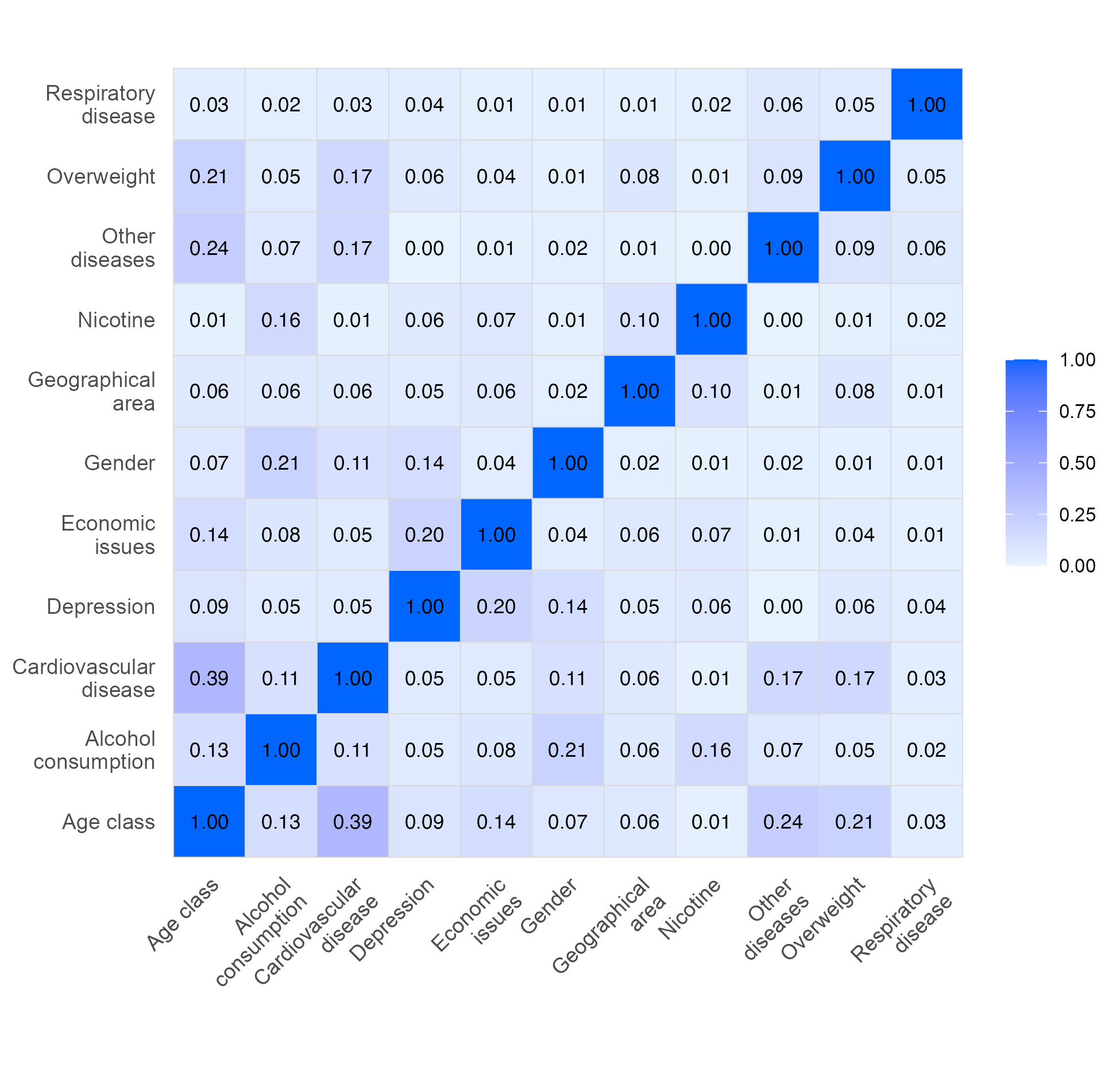

Supplement: S1 Fig — (TIF) [file pone.0342466.s003.tif]

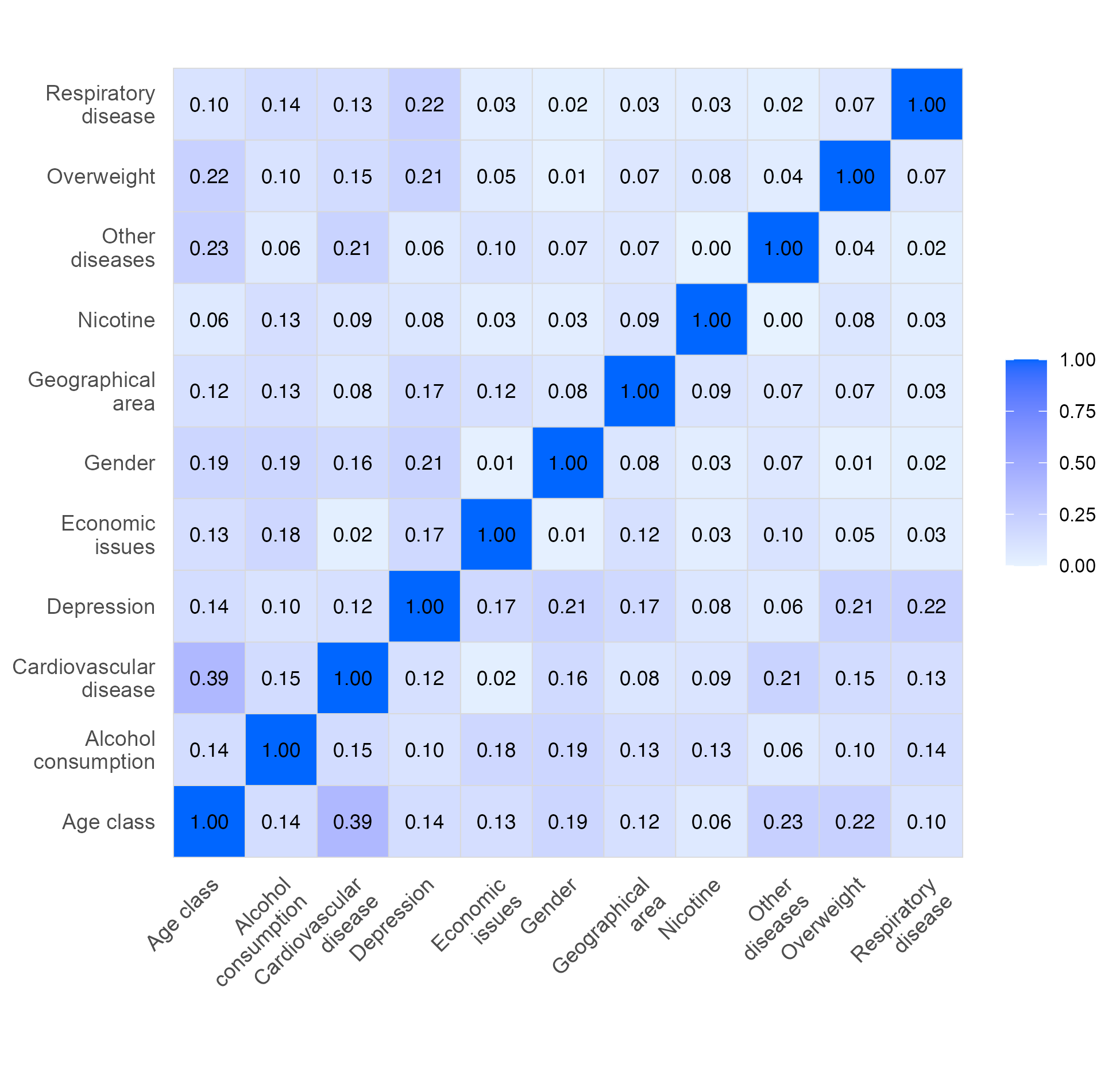

Supplement: S2 Fig — (TIF) [file pone.0342466.s004.tif]
