# Supplementary material for: Treatments for COVID-19 and acute respiratory infections are associated with gender and comorbidities in an Italian online survey
Source: PLoS One. 2026 Feb 17;21(2):e0342466. doi: 10.1371/journal.pone.0342466 (PMC12912575; doi:10.1371/journal.pone.0342466)
Supplement: S1 Table — n.s. = non-significant p-values at level 0.05. (DOCX) [file pone.0342466.s005.docx]

| Variable | Painkillers  (incl. NSAIDs) | Paracetamol | Antibiotics | Macrolide antibiotics | Food suppl. | Ibuprofen | Steroids |
| --- | --- | --- | --- | --- | --- | --- | --- |
| Sex | n.s. | n.s. | n.s. | n.s. | n.s. | 0.021 | n.s. |
| Age class | n.s. | n.s. | 0.002 | n.s. | n.s. | n.s. | n.s. |
| Geographical area | 0.019 | n.s. | n.s. | n.s. | n.s. | n.s. | 0.007 |
| Economic issues | n.s. | n.s. | n.s. | n.s. | n.s. | n.s. | n.s. |
| Weight class | n.s. | 0.039 | 0.026 | 0.032 | n.s. | n.s. | n.s. |
| Respiratory diseases | n.s. | n.s. | n.s. | n.s. | n.s. | n.s. | 0.009 |
| Cardiovascular diseases | n.s. | n.s. | 0.026 | 0.013 | n.s. | n.s. | n.s. |
| Other comorbidities | n.s. | n.s. | 0.010 | n.s. | n.s. | n.s. | n.s. |
| Depression severity | n.s. | n.s. | n.s. | n.s. | n.s. | 0.032 | n.s. |
| Alcohol | n.s. | n.s. | n.s. | n.s. | n.s. | n.s. | n.s. |
| Nicotine | n.s. | n.s. | n.s. | n.s. | n.s. | n.s. | n.s. |
