# Supplementary material for: Treatments for COVID-19 and acute respiratory infections are associated with gender and comorbidities in an Italian online survey
Source: PLoS One. 2026 Feb 17;21(2):e0342466. doi: 10.1371/journal.pone.0342466 (PMC12912575; doi:10.1371/journal.pone.0342466)
Supplement: S2 Table — n.s. = non-significant p-values at level 0.05. (DOCX) [file pone.0342466.s006.docx]

| Variable | Painkillers  (incl. NSAIDs) | Paracetamol | Antibiotics | Aspirin | Food supplements | Ibuprofen | Syrups chesty cough | Home remedies | Nose Sprays | Vitamin suppl. |
| --- | --- | --- | --- | --- | --- | --- | --- | --- | --- | --- |
| Gender | n.s. | n.s. | n.s. | 0.025 | 0.003 | 0.024 | n.s. | <0.001 | 0.016 | 0.013 |
| Age class | n.s. | n.s. | <0.001 | n.s. | n.s. | 0.035 | n.s. | n.s. | n.s. | n.s. |
| Geographical area | 0.003 | <0.001 | n.s. | n.s. | n.s. | n.s. | n.s. | n.s. | n.s. | n.s. |
| Economic issues | n.s. | n.s. | n.s. | n.s. | n.s. | n.s. | n.s. | n.s. | 0.049 | n.s. |
| Overweight | 0.030 | <0.001 | 0.003 | n.s. | n.s. | n.s. | n.s. | n.s. | 0.030 | n.s. |
| Respiratory diseases | 0.031 | n.s. | <0.001 | n.s. | n.s. | 0.034 | n.s. | n.s. | 0.020 | n.s. |
| Cardiovascular diseases | n.s. | n.s. | 0.012 | n.s. | n.s. | n.s. | n.s. | n.s. | n.s. | n.s. |
| Other comorbidities | n.s. | 0.024 | 0.017 | n.s. | n.s. | n.s. | n.s. | n.s. | n.s. | n.s. |
| Depression severity | 0.003 | 0.011 | n.s. | n.s. | n.s. | 0.001 | 0.036 | n.s. | 0.015 | n.s. |
| Alcohol | n.s. | n.s. | n.s. | n.s. | n.s. | n.s. | 0.005 | n.s. | n.s. | n.s. |
| Nicotine | n.s. | n.s. | n.s. | n.s. | n.s. | n.s. | n.s. | n.s. | n.s. | n.s. |
