# Supplementary material for: Treatments for COVID-19 and acute respiratory infections are associated with gender and comorbidities in an Italian online survey
Source: PLoS One. 2026 Feb 17;21(2):e0342466. doi: 10.1371/journal.pone.0342466 (PMC12912575; doi:10.1371/journal.pone.0342466)
Supplement: S3 Table — (DOCX) [file pone.0342466.s007.docx]

**Supplementary Table 3**

| **Treatments taken for COVID-19** |  |  |  |  |  |  |
| --- | --- | --- | --- | --- | --- | --- |
| **1. MODERN MEDICINE** | **Total** | **%** | **Total ♀** | **%** | **Total ♂** | **%** |
| **Painkillers (incl. NSAIDs)** | 206 | 52,7 | 116 | 51,3 | 90 | 55,2 |
| Paracetamol (acetaminophen) | 155 | 39,6 | 86 | 38,1 | 69 | 42,3 |
| Ibuprofen | 61 | 15,6 | 42 | 18,6 | 19 | 11,7 |
| Aspirin (ASA) | 32 | 8,2 | 17 | 7,5 | 15 | 9,2 |
| Throat sprays | 17 | 4,3 | 11 | 4,9 | 6 | 3,7 |
| Other analgesics (Naproxen, ketoprofen) | 10 | 2,6 | 4 | 1,8 | 6 | 3,7 |
|  |  |  |  |  |  |  |
| **Antibiotics** | 104 | 26,6 | 61 | 27 | 42 | 25,8 |
| Macrolides (mycines) | 65 | 16,6 | 42 | 18,6 | 23 | 14,1 |
| Amoxicillin + clavulanic acid | 29 | 7,4 | 15 | 6,6 | 13 | 8 |
| Other antibiotics (did not remember name) | 9 | 2,3 | 3 | 1,3 | 6 | 3,7 |
| Cephalosporins | 4 | 1 | 3 | 1,3 | 1 | 0,6 |
| Penicillins | 2 | 0,5 | 1 | 0,4 | 1 | 0,6 |
| Floxacin | 1 | 0,3 | 0 | 0 | 1 | 0,6 |
| Flucloxacillin | 1 | 0,3 | 0 | 0 | 1 | 0,6 |
|  |  |  |  |  |  |  |
| **Syrups chesty cough (Syrups, drops, or lozenges)** | 29 | 7,4 | 14 | 6,2 | 15 | 9,2 |
|  |  |  |  |  |  |  |
| **Syrups dry cough (Syrups, drops, or lozenges)** | 18 | 4,6 | 14 | 6,2 | 4 | 2,5 |
|  |  |  |  |  |  |  |
| **Nose sprays (Steroids, isotonic, hypertonic, adrenergic agonists)** | 29 | 7,4 | 18 | 8 | 11 | 6,7 |
| Nasal decongestant (e.g., Sudafed^®^, Otrivine^®^) | 8 | 2 | 3 | 1,3 | 5 | 3,1 |
| Isotonic (e.g., Sterimar isotonic nasal spray) | 7 | 1,8 | 4 | 1,8 | 3 | 1,8 |
| Steroid nose spray (e.g., Beconase^®^, Flixonase^®^, Nasonex^®^) | 5 | 1,3 | 2 | 0,9 | 3 | 1,8 |
| Hypertonic saline spray (e.g., Sterimar congestion relief sea water nasal spray) | 2 | 0,5 | 1 | 0,4 | 1 | 0,6 |
| Nose spray (did not remember name) | 4 | 1 | 3 | 1,3 | 1 | 0,6 |
|  |  |  |  |  |  |  |
| **Steroids (tablets, nose sprays)** | 57 | 14,6 | 28 | 12,4 | 28 | 17,2 |
| Steroid nose spray (e.g., Beconase^®^, Flixonase^®^, Nasonex^®^) | 5 | 1,3 | 2 | 0,9 | 3 | 1,8 |
|  |  |  |  |  |  |  |
| **Inhalers (LABA, SABA, corticosteroids, anticholinergics)** | 19 | 4,9 | 11 | 4,9 | 8 | 4,9 |
| Green/pink (Long-acting β2 adrenergic receptor agonist (LABA)) | 9 | 2,3 | 5 | 2,2 | 4 | 2,5 |
| Pink (Long-acting β2 adrenergic receptor agonist (LABA)) | 6 | 1,5 | 3 | 1,3 | 3 | 1,8 |
| Green (Long-acting β2 adrenergic receptor agonist (LABA)) | 3 | 0,8 | 2 | 0,9 | 1 | 0,6 |
|  |  |  |  |  |  |  |
| Blue (Short-acting β2 adrenergic receptor agonist (SABA)) | 7 | 1,8 | 4 | 1,8 | 3 | 1,8 |
|  |  |  |  |  |  |  |
| White (Anticholinergics) | 4 | 1 | 2 | 0,9 | 2 | 1,2 |
|  |  |  |  |  |  |  |
| Brown (Corticosteroids) | 1 | 0,3 | 1 | 0,4 | 0 | 0 |
|  |  |  |  |  |  |  |
| Inhaler (did not remember name) | 3 | 0,8 | 2 | 0,9 | 1 | 0,6 |
|  |  |  |  |  |  |  |
| **Antihistamines** | 9 | 2,3 | 6 | 2,7 | 3 | 1,8 |
|  |  |  |  |  |  |  |
| **Hydroxychloroquine** | 10 | 2,6 | 5 | 2,2 | 5 | 3,1 |
| **Chloroquine** | 4 | 1 | 1 | 0,4 | 3 | 1,8 |
|  |  |  |  |  |  |  |
|  |  |  |  |  |  |  |
| **2. CAM SUPPLEMENTS** | **Total** | **%** | **Total ♀** | **%** | **Total ♂** | **%** |
| **Food supplements (vitamins, minerals, amino-acids, omega-3)** | 61 | 15,6 | 43 | 19 | 18 | 11 |
| **Vitamin supplementation** | 48 | 12,3 | 32 | 14,2 | 16 | 9,8 |
| Vitamin C | 39 | 10 | 26 | 11,5 | 13 | 8 |
| Vitamin D | 29 | 7,4 | 21 | 9,3 | 8 | 4,9 |
| Vitamin B12 | 13 | 3,3 | 10 | 4,4 | 3 | 1,8 |
| Vitamin E | 7 | 1,8 | 5 | 2,2 | 2 | 1,2 |
| Vitamin A | 6 | 1,5 | 4 | 1,8 | 2 | 1,2 |
| Multi Vitamins | 30 | 7,7 | 21 | 9,3 | 9 | 5,6 |
| Vitamin mono (did not remember name) | 13 | 3,3 | 10 | 4,4 | 3 | 1,8 |
|  |  |  |  |  |  |  |
| **Mineral supplementation** | 14 | 3,6 | 10 | 4,4 | 4 | 2,5 |
| Magnesium | 11 | 2,8 | 8 | 3,5 | 3 | 1,8 |
| Zinc | 7 | 1,8 | 5 | 2,2 | 2 | 1,2 |
| Calcium | 6 | 1,5 | 3 | 1,3 | 3 | 1,8 |
| Selenium | 5 | 1,3 | 3 | 1,3 | 2 | 1,2 |
| Copper | 2 | 0,5 | 0 | 0 | 2 | 1,2 |
| Chromium | 2 | 0,5 | 0 | 0 | 2 | 1,2 |
| Mineral (did not remember name) | 1 | 0,3 | 0 | 0 | 1 | 0,6 |
|  |  |  |  |  |  |  |
| **Omega-3 fatty acids (Cod liver oil, linseed oil, algal oils)** | 3 | 0,8 | 3 | 1,3 | 0 | 0 |
|  |  |  |  |  |  |  |
| **Amino acids** | 4 | 1 | 4 | 1,8 | 0 | 0 |
|  |  |  |  |  |  |  |
| **Enzyme supplements** | 1 | 0,3 | 1 | 0,5 | 0 | 0 |
|  |  |  |  |  |  |  |
|  |  |  |  |  |  |  |
| **3. HERBAL PREPARATIONS and HOME REMEDIES** | **Total** | **%** | **Total ♀** | **%** | **Total ♂** | **%** |
| **Home remedies (e.g., ginger and lemon, inhalation, nasal rinse)** | 26 | 6,6 | 19 | 8,4 | 7 | 4,3 |
| **Nasal rinse** | 7 | 1,8 | 5 | 2,2 | 2 | 1,2 |
| **Steam Inhalation** | 7 | 1,8 | 6 | 2,7 | 1 | 0,6 |
|  |  |  |  |  |  |  |
| **Herbal medicines (teas, capsules, syrups, drops, extracts)** | 18 | 4,6 | 13 | 5,8 | 5 | 3,1 |
| Traditional Western medicine and phytotherapy | 11 | 2,8 | 8 | 3,5 | 3 | 1,8 |
| Ayurvedic medicine | 2 | 0,5 | 2 | 0,9 | 0 | 0 |
| Traditional Chinese medicine | 1 | 0,3 | 0 | 0 | 1 | 0,6 |
|  |  |  |  |  |  |  |
| **Preparations** |  |  |  |  |  |  |
| **Herbal teas** | 5 | 1,3 | 4 | 1,8 | 1 | 0,6 |
| **Herbal syrups or drops** | 6 | 1,5 | 5 | 2,2 | 1 | 0,6 |
| **Herbal capsules, pills, or tablets** | 5 | 1,3 | 2 | 0,9 | 3 | 1,8 |
| **Nose sprays** | 1 | 0,3 | 0 | 0 | 1 | 0,6 |
|  |  |  |  |  |  |  |
| **GINGER (*Zingiber officinale* Roscoe)** |  |  |  |  |  |  |
| **Infusion, capsules, pills, tablets, drops or syrup** | 18 | 4,6 | 12 | 5,3 | 6 | 3,7 |
| Ginger and honey tea | 14 | 3,6 | 9 | 4 | 5 | 3,1 |
| Herbal tea | 3 | 0,8 | 2 | 0,9 | 1 | 0,6 |
| Capsules, pills, or tablets | 1 | 0,3 | 1 | 0,4 | 0 | 0 |
| Drops or syrup | 1 | 0,3 | 1 | 0,4 | 0 | 0 |
|  |  |  |  |  |  |  |
| **LEMON (*Citrus limon* L.)** |  |  |  |  |  |  |
| **Infusion, capsules, pills, tablets, drops or syrup** | 16 | 4,1 | 10 | 4,4 | 6 | 3,7 |
| Lemon and honey (tea) | 9 | 2,3 | 7 | 3,1 | 2 | 1,2 |
| Lemon juice | 4 | 1 | 3 | 1,3 | 1 | 0,6 |
| Capsules, pills, or tablets | 1 | 0,3 | 1 | 0,4 | 0 | 0 |
|  |  |  |  |  |  |  |
| **HONEY** |  |  |  |  |  |  |
| Honey and ginger tea | 14 | 3,6 | 9 | 4 | 5 | 3,1 |
| Honey in herbal tea | 13 | 3,3 | 9 | 4 | 4 | 2,5 |
| Honey and lemon (tea) | 9 | 2,3 | 7 | 3,1 | 2 | 1,2 |
| Bee products (mainly honey) | 6 | 1,5 | 4 | 1,8 | 2 | 1,2 |
|  |  |  |  |  |  |  |
| **TURMERIC (*Curcuma* sp.)** |  |  |  |  |  |  |
| Infusion, capsules, pills, tablets, drops, syrup, food | 7 | 1,8 | 5 | 2,2 | 2 | 1,2 |
| Turmeric tea | 2 | 0,5 | 2 | 0,9 | 0 | 0 |
|  |  |  |  |  |  |  |
| **GARLIC (*Allium sativum* L.)** |  |  |  |  |  |  |
| Food, capsules, pills, tablets | 3 | 0,8 | 1 | 0,4 | 2 | 1,2 |
|  |  |  |  |  |  |  |
| **ONION (*Allium cepa* L.)** |  |  |  |  |  |  |
| Food, capsules, pills, tablets | 3 | 0,8 | 2 | 0,9 | 1 | 0,6 |
|  |  |  |  |  |  |  |
| **ECHINACEA (*Echinaceae* sp.)** |  |  |  |  |  |  |
| **Infusion, capsules, pills, tablets, drops or syrup** | 4 | 1 | 1 | 0,4 | 3 | 1,8 |
| Drops or syrup | 2 | 0,5 | 1 | 0,4 | 1 | 0,6 |
| Capsules, pills, or tablets | 2 | 0,5 | 0 | 0 | 2 | 1,2 |
|  |  |  |  |  |  |  |
| **THYME (*Thymus* sp.)** |  |  |  |  |  |  |
| **Infusion, capsules, pills, tablets, drops or syrup** | 1 | 0,3 | 1 | 0,4 | 0 | 0 |
| Herbal tea | 1 | 0,3 | 1 | 0,4 | 0 | 0 |
|  |  |  |  |  |  |  |
| **ELDERFLOWER (*Sambucus nigra* L.)** |  |  |  |  |  |  |
| **Infusion, capsules, pills, tablets, drops or syrup** | 1 | 0,3 | 1 | 0,4 | 0 | 0 |
| Herbal tea | 1 | 0,3 | 1 | 0,4 | 0 | 0 |
|  |  |  |  |  |  |  |
|  |  |  |  |  |  |  |
| **Various herbal preparations** |  |  |  |  |  |  |
| Multi-compound (herbal tea) | 2 | 0,5 | 1 | 0,4 | 1 | 0,6 |
| Lime (*Tilia* sp., herbal tea) | 2 | 0,5 | 2 | 0,9 | 0 | 0 |
| Apple cider / vinegar | 2 | 0,5 | 2 | 0,9 | 0 | 0 |
| Hibiscus (*Hibiscus sabdariffa* L., herbal tea) | 1 | 0,3 | 1 | 0,4 | 0 | 0 |
| *Mentha* sp. (capsules, pills, or tablets) | 1 | 0,3 | 1 | 0,4 | 0 | 0 |
| Turnip (*Brassica rapa* L., syrup) | 1 | 0,3 | 0 | 0 | 1 | 0,6 |
| Gelodurat essential oil capsules (*Eucalyptus* sp., *Myrtus* sp., *Citrus* spp.) | 1 | 0,3 | 1 | 0,5 | 0 | 0 |
|  |  |  |  |  |  |  |
| **TCM treatments and herbal preparations** | 1 | 0,3 | 0 | 0 | 1 | 0,6 |
|  |  |  |  |  |  |  |
| **Ayurvedic preparations** | 2 | 0,5 | 2 | 0,9 | 0 | 0 |
| Anu thailam (multi compound nasal oil) | 1 | 0,3 | 1 | 0,4 | 0 | 0 |
| Tulsi leaves (*Ocimum tenuiflorum* L.) | 1 | 0,3 | 1 | 0,4 | 0 | 0 |
|  |  |  |  |  |  |  |
| **Homeopathy** | 8 | 2 | 7 | 3,1 | 1 | 0,6 |
| Bronchi plantago | 1 | 0,3 | 1 | 0,4 | 0 | 0 |
|  |  |  |  |  |  |  |
| **ESSENTIAL OILS (inhalation)** | 5 | 1,3 | 3 | 1,3 | 2 | 1,2 |
|  |  |  |  |  |  |  |
| **Eucalyptus (*Eucalyptus* sp.)** | 4 | 1 | 3 | 1,3 | 1 | 0,6 |
|  |  |  |  |  |  |  |
| **Tea Tree (*Melaleuca alternifolia* (Maiden & Betche) Cheel)** | 2 | 0,5 | 1 | 0,4 | 1 | 0,6 |
|  |  |  |  |  |  |  |
| **Vicks First Defense^®^ inhaler^®^ nasal stick^®^ (Menthol, camphor, eucalyptol)** | 3 | 0,8 | 1 | 0,4 | 2 | 1,2 |
|  |  |  |  |  |  |  |
| **Olbas (*Melaleuca leucadendra* (L.) L., *Syzygium aromaticum* (L.) Merr. & L.M. Perry, *Eucalyptus* sp., *Juniperus* sp., levomenthol, methyl salicylate, *Mentha* sp.)** | 1 | 0,3 | 1 | 0,4 | 0 | 0 |
|  |  |  |  |  |  |  |
| **Oregano (*Origanum vulgare* L.)** | 1 | 0,3 | 0 | 0 | 1 | 0,6 |
|  |  |  |  |  |  |  |
| **Ravintsara (*Cinnamomum camphora* (L.) J. Presl)** | 1 | 0,3 | 0 | 0 | 1 | 0,6 |
|  |  |  |  |  |  |  |
|  |  |  |  |  |  |  |
| **DIET / FOOD** | **Total** | **%** | **Total ♀** | **%** | **Total ♂** | **%** |
| **Special foods (Dairy-free, gluten-free probiotics, honey, fruits, soups, spices)** | 16 | 4,1 | 11 | 4,9 | 5 | 3,1 |
| **Special diets (gluten-free, dairy-free)** | 2 | 0,5 | 2 | 0,9 | 0 | 0 |
| Gluten-free | 1 | 0,3 | 1 | 0,4 | 0 | 0 |
|  |  |  |  |  |  |  |
| **Fruits and vegetables** | 7 | 1,8 | 4 | 1,8 | 3 | 1,8 |
| **Soups** | 3 | 0,8 | 1 | 0,4 | 2 | 1,2 |
| **Probiotics suppl. (e.g., Yakult, Actimel)** | 5 | 1,3 | 3 | 1,3 | 2 | 1,2 |
| **Spices** | 4 | 1 | 2 | 0,9 | 2 | 1,2 |
| **Kombucha, kefir** | 2 | 0,5 | 1 | 0,4 | 1 | 0,6 |
| **Seaweed** | 1 | 0,3 | 0 | 0 | 1 | 0,6 |
|  |  |  |  |  |  |  |
| **EXERCISE OR ACTIVITIES** | 8 | 2 | 5 | 2,2 | 3 | 1,8 |
| Walking, hiking | 2 | 0,5 | 1 | 0,4 | 1 | 0,6 |
| Sport activities | 3 | 0,8 | 2 | 0,9 | 1 | 0,6 |
| Meditation, mindfulness | 1 | 0,3 | 1 | 0,4 | 0 | 0 |
| Yoga | 1 | 0,3 | 1 | 0,4 | 0 | 0 |
| Physiotherapy | 1 | 0,3 | 0 | 0 | 1 | 0,6 |
