# Supplementary material for: Treatments for COVID-19 and acute respiratory infections are associated with gender and comorbidities in an Italian online survey
Source: PLoS One. 2026 Feb 17;21(2):e0342466. doi: 10.1371/journal.pone.0342466 (PMC12912575; doi:10.1371/journal.pone.0342466)
Supplement: S4 Table — (DOCX) [file pone.0342466.s008.docx]

| **Treatments taken for acute respiratory infections (ARIs)** |  |  |  |  |  |  |
| --- | --- | --- | --- | --- | --- | --- |
| **1. MODERN MEDICINE** | **Total** | **%** | **Total ♀** | **%** | **Total ♂** | **%** |
| **Painkillers (incl. NSAIDs)** | 1084 | 44 | 662 | 45,5 | 418 | 41,8 |
| Paracetamol (acetaminophen) | 751 | 30,5 | 458 | 31,5 | 289 | 28,9 |
| Ibuprofen | 342 | 13,9 | 222 | 15,3 | 120 | 12 |
| Aspirin (ASA) | 219 | 8,9 | 113 | 7,8 | 104 | 10,4 |
| Throat sprays | 196 | 8 | 127 | 8,7 | 68 | 6,8 |
| Other analgesics (Naproxen, ketoprofen) | 102 | 4,1 | 55 | 3,8 | 47 | 4,7 |
|  |  |  |  |  |  |  |
| **Antibiotics** | 380 | 15,4 | 226 | 15,5 | 152 | 15,2 |
| Amoxicillin + clavulanic acid | 152 | 6,2 | 83 | 5,7 | 68 | 6,8 |
| Macrolides (mycines) | 127 | 5,2 | 81 | 5,6 | 45 | 4,5 |
| Other antibiotics (did not remember name) | 66 | 2,7 | 39 | 2,7 | 27 | 2,7 |
| Cephalosporins | 21 | 0,9 | 15 | 1 | 6 | 0,6 |
| Floxacin | 10 | 0,4 | 5 | 0,3 | 5 | 0,5 |
| Flucloxacillin | 3 | 0,1 | 1 | 0,1 | 2 | 0,2 |
| Penicillins | 4 | 0,2 | 2 | 0,1 | 2 | 0,2 |
| Cyclines | 1 | 0 | 1 | 0,1 | 0 | 0 |
|  |  |  |  |  |  |  |
| **Syrups chesty cough (Syrups, drops, or lozenges)** | 243 | 9,9 | 154 | 10,6 | 88 | 8,8 |
|  |  |  |  |  |  |  |
| **Syrups dry cough (Syrups, drops, or lozenges)** | 150 | 6,1 | 109 | 7,5 | 41 | 4,1 |
|  |  |  |  |  |  |  |
| **Nose sprays (Steroids, isotonic, hypertonic, adrenergic agonists, essential oils)** | 242 | 9,8 | 161 | 11,1 | 81 | 8,1 |
| Nasal decongestant (e.g., Sudafed^®^, Otrivine^®^) | 55 | 2,2 | 35 | 2,4 | 20 | 2 |
| Isotonic (e.g., Sterimar isotonic nasal spray) | 44 | 1,8 | 32 | 2,2 | 12 | 1,2 |
| Steroid nose spray (e.g., Beconase^®^, Flixonase^®^, Nasonex^®^) | 32 | 1,3 | 17 | 1,2 | 15 | 1,5 |
| Hypertonic saline spray (e.g. Sterimar congestion relief sea water nasal spray) | 25 | 1 | 16 | 1,1 | 9 | 0,9 |
| Nose spray (did not remember name) | 59 | 2,4 | 36 | 2,5 | 23 | 2,3 |
|  |  |  |  |  |  |  |
| **Steroids (tablets, nose sprays)** | 152 | 6,2 | 97 | 6,7 | 54 | 5,4 |
| Steroid nose spray (e.g., Beconase^®^, Flixonase^®^, Nasonex^®^) | 32 | 1,3 | 17 | 1,2 | 15 | 1,5 |
|  |  |  |  |  |  |  |
| **Inhalers (LABA, SABA, corticosteroids, anticholinergics)** | 94 | 3,8 | 61 | 4,2 | 33 | 3,3 |
| Green/pink (Long-acting β2 adrenergic receptor agonist (LABA)) | 42 | 1,7 | 29 | 2 | 13 | 1,3 |
| Pink (Long-acting β2 adrenergic receptor agonist (LABA)) | 26 | 1,1 | 15 | 1 | 11 | 1,1 |
| Green (Long-acting β2 adrenergic receptor agonist (LABA)) | 18 | 0,7 | 15 | 1 | 3 | 0,3 |
|  |  |  |  |  |  |  |
| Blue (Short-acting β2 adrenergic receptor agonist (SABA)) | 24 | 1 | 15 | 1 | 9 | 0,9 |
|  |  |  |  |  |  |  |
| White (Anticholinergics) | 16 | 0,6 | 10 | 0,7 | 6 | 0,6 |
|  |  |  |  |  |  |  |
| Brown (Corticosteroids) | 4 | 0,2 | 3 | 0,2 | 1 | 0,1 |
|  |  |  |  |  |  |  |
| Inhaler (did not remember name) | 16 | 0,6 | 7 | 0,5 | 9 | 0,9 |
|  |  |  |  |  |  |  |
| **Antihistamines** | 63 | 2,6 | 35 | 2,4 | 28 | 2,8 |
|  |  |  |  |  |  |  |
| **Hydroxychloroquine** | 16 | 0,6 | 7 | 0,5 | 9 | 0,9 |
| **Chloroquine** | 6 | 0,2 | 2 | 0,1 | 4 | 0,4 |
|  |  |  |  |  |  |  |
|  |  |  |  |  |  |  |
| **2. CAM SUPPLEMENTS** | **Total** | **%** | **Total ♀** | **%** | **Total ♂** | **%** |
| **Food supplements (Food supplements: vitamins, minerals, amino-acids, omega-3)** | 280 | 11,4 | 189 | 13 | 91 | 9,1 |
| **Vitamin supplementation** | 212 | 8,6 | 143 | 9,8 | 69 | 6,9 |
| Vitamin C | 165 | 6,7 | 117 | 8 | 48 | 4,8 |
| Vitamin D | 114 | 4,6 | 77 | 5,3 | 37 | 3,7 |
| Vitamin B12 | 66 | 2,7 | 43 | 3 | 23 | 2,3 |
| Vitamin A | 36 | 1,5 | 27 | 1,9 | 9 | 0,9 |
| Vitamin E | 29 | 1,2 | 20 | 1,4 | 9 | 0,9 |
| Multi Vitamins | 143 | 5,8 | 91 | 6,2 | 52 | 5,2 |
| Vitamin mono (did not remember name) | 60 | 2,4 | 45 | 3,1 | 15 | 1,5 |
|  |  |  |  |  |  |  |
| **Mineral supplementation** | 66 | 2,7 | 41 | 2,8 | 25 | 2,5 |
| Magnesium | 51 | 2,1 | 33 | 2,3 | 18 | 1,8 |
| Zinc | 28 | 1,1 | 16 | 1,1 | 12 | 1,2 |
| Calcium | 18 | 0,7 | 12 | 0,8 | 6 | 0,6 |
| Selenium | 12 | 0,5 | 10 | 0,7 | 2 | 0,2 |
| Copper | 5 | 0,2 | 1 | 0,1 | 4 | 0,4 |
| Chromium | 3 | 0,1 | 1 | 0,1 | 2 | 0,2 |
| Mineral (did not remember name) | 10 | 0,4 | 6 | 0,4 | 4 | 0,4 |
|  |  |  |  |  |  |  |
| **Omega-3 fatty acids (Cod liver oil, linseed oil, algal oils)** | 34 | 1,4 | 20 | 1,4 | 14 | 1,4 |
|  |  |  |  |  |  |  |
| **Amino acids** | 15 | 0,6 | 10 | 0,7 | 5 | 0,5 |
|  |  |  |  |  |  |  |
| **Enzyme supplements** | 2 | 0,09 | 1 | 0,08 | 1 | 0,11 |
|  |  |  |  |  |  |  |
|  |  |  |  |  |  |  |
| **3. HERBAL PREPARATIONS and HOME REMEDIES** | **Total** | **%** | **Total ♀** | **%** | **Total ♂** | **%** |
| **Home remedies (e.g., ginger and lemon, inhalation, nasal rinse)** | 226 | 9,2 | 159 | 10,9 | 65 | 6,5 |
| **Nasal rinse** | 81 | 3,3 | 55 | 3,8 | 26 | 2,6 |
| **Steam Inhalation** | 79 | 3,2 | 60 | 4,1 | 19 | 1,9 |
|  |  |  |  |  |  |  |
| **Herbal medicines (teas, capsules, syrups, drops, extracts)** | 150 | 6,1 | 113 | 7,8 | 35 | 3,5 |
| Traditional Western medicine and phytotherapy | 92 | 3,7 | 72 | 5 | 19 | 1,9 |
| Traditional Chinese medicine | 4 | 0,2 | 2 | 0,1 | 2 | 0,2 |
| Ayurvedic medicine | 4 | 0,2 | 4 | 0,3 | 0 | 0 |
|  |  |  |  |  |  |  |
| **Preparations** |  |  |  |  |  |  |
| **Herbal teas** | 58 | 2,4 | 47 | 3,2 | 10 | 1 |
| **Herbal syrups or drops** | 39 | 1,6 | 32 | 2,2 | 6 | 0,6 |
| **Herbal capsules, pills, or tablets** | 34 | 1,4 | 25 | 1,7 | 8 | 0,8 |
| **Nose sprays** | 16 | 0,6 | 11 | 0,8 | 5 | 0,5 |
| **Ointments or lotion for external application** | 5 | 0,2 | 5 | 0,3 | 0 | 0 |
|  |  |  |  |  |  |  |
| **GINGER (*Zingiber officinale* Roscoe)** |  |  |  |  |  |  |
| **Infusion, capsules, pills, tablets, drops or syrup** | 155 | 6,3 | 107 | 7,4 | 46 | 4,6 |
| Ginger and honey tea | 130 | 5,3 | 89 | 6,1 | 39 | 3,9 |
| Herbal tea | 36 | 1,5 | 27 | 1,9 | 8 | 0,8 |
| Capsules, pills, or tablets | 8 | 0,3 | 6 | 0,4 | 2 | 0,2 |
| Drops or syrup | 3 | 0,1 | 3 | 0,2 | 0 | 0 |
|  |  |  |  |  |  |  |
| **LEMON (*Citrus limon* L.)** |  |  |  |  |  |  |
| **Infusion, capsules, pills, tablets, drops or syrup** | 120 | 4,9 | 83 | 5,7 | 35 | 3,5 |
| Lemon and honey (tea) | 78 | 3,2 | 56 | 3,9 | 21 | 2,1 |
| Lemon juice | 40 | 1,6 | 26 | 1,8 | 13 | 1,3 |
| Capsules, pills, or tablets | 6 | 0,2 | 5 | 0,3 | 1 | 0,1 |
|  |  |  |  |  |  |  |
| **HONEY** |  |  |  |  |  |  |
| Honey and ginger tea | 130 | 5,3 | 89 | 6,1 | 39 | 3,9 |
| Honey in herbal tea | 114 | 4,6 | 78 | 5,4 | 35 | 3,5 |
| Honey and lemon (tea) | 78 | 3,2 | 56 | 3,9 | 21 | 2,1 |
| Bee products (mainly honey) | 57 | 2,3 | 36 | 2,5 | 21 | 2,1 |
|  |  |  |  |  |  |  |
| **TURMERIC (*Curcuma* sp.)** |  |  |  |  |  |  |
| Infusion, capsules, pills, tablets, drops, syrup, food | 39 | 1,6 | 28 | 1,9 | 10 | 1 |
| Turmeric tea | 20 | 0,8 | 15 | 1 | 4 | 0,4 |
|  |  |  |  |  |  |  |
| **GARLIC (*Allium sativum* L.)** |  |  |  |  |  |  |
| Food, capsules, pills, tablets | 30 | 1,2 | 20 | 1,4 | 10 | 1 |
| Garlic and onion soup | 4 | 0,2 | 2 | 0,1 | 2 | 0,2 |
| Capsules, pills, or tablets | 4 | 0,2 | 4 | 0,3 | 0 | 0 |
|  |  |  |  |  |  |  |
| **ONION (*Allium cepa* L.)** |  |  |  |  |  |  |
| Food, syrup | 28 | 1,1 | 19 | 1,3 | 9 | 0,9 |
| Onion and garlic soup | 4 | 0,2 | 2 | 0,1 | 2 | 0,2 |
| Syrup | 1 | 0 | 0 | 0 | 1 | 0,1 |
|  |  |  |  |  |  |  |
| **ECHINACEA (*Echinaceae* sp.)** |  |  |  |  |  |  |
| **Infusion, capsules, pills, tablets, drops or syrup** | 35 | 1,4 | 27 | 1,9 | 7 | 0,7 |
| Drops or syrup | 15 | 0,6 | 12 | 0,8 | 2 | 0,2 |
| Capsules, pills, or tablets | 12 | 0,5 | 8 | 0,6 | 4 | 0,4 |
|  |  |  |  |  |  |  |
| **THYME (*Thymus* sp.)** |  |  |  |  |  |  |
| **Infusion, capsules, pills, tablets, drops or syrup** | 18 | 0,7 | 14 | 1 | 3 | 0,3 |
| Herbal tea | 15 | 0,6 | 12 | 0,8 | 2 | 0,2 |
|  |  |  |  |  |  |  |
| **ELDERFLOWER (*Sambucus nigra* L.)** |  |  |  |  |  |  |
| **Infusion, capsules, pills, tablets, drops or syrup** | 14 | 0,6 | 13 | 0,9 | 1 | 0,1 |
| Herbal tea | 7 | 0,3 | 6 | 0,4 | 1 | 0,1 |
| Capsules, pills, or tablets | 2 | 0,1 | 2 | 0,1 | 0 | 0 |
| Drops or syrup | 1 | 0 | 1 | 0,1 | 0 | 0 |
|  |  |  |  |  |  |  |
| **GINSENG (*Panax ginseng* C.A. Mey)** |  |  |  |  |  |  |
| **Infusion, capsules, pills, tablets, drops or syrup** | 2 | 0,1 | 2 | 0,1 | 0 | 0 |
| Capsules, pills, or tablets | 1 | 0 | 1 | 0,1 | 0 | 0 |
| Drops or syrup | 1 | 0 | 1 | 0,1 | 0 | 0 |
|  |  |  |  |  |  |  |
| **Various herbal preparations** |  |  |  |  |  |  |
| Herbal tea (did not remember name) | 24 | 1 | 17 | 1,2 | 7 | 0,7 |
| Multi-compound (herbal tea) | 21 | 0,9 | 15 | 1 | 6 | 0,6 |
| Lime (*Tilia* sp., herbal tea) | 11 | 0,4 | 10 | 0,7 | 1 | 0,1 |
| Apple cider / vinegar | 10 | 0,4 | 9 | 0,6 | 1 | 0,1 |
| Sage (*Salvia officinalis* L., herbal tea) | 9 | 0,4 | 8 | 0,6 | 1 | 0,1 |
| Drops or syrup (did not remember name) | 8 | 0,3 | 7 | 0,5 | 1 | 0,1 |
| Hibiscus (*Hibiscus sabdariffa* L., herbal tea) | 5 | 0,2 | 4 | 0,3 | 1 | 0,1 |
| Capsules, pills, or tablets (did not remember name) | 3 | 0,13 | 3 | 0,23 | 0 | 0 |
| Creat (*Andrographis paniculata* (Burm.f.) Wall. ex Nees,  Capsules, pills, or tablets) | 3 | 0,13 | 2 | 0,15 | 0 | 0 |
| *Mentha* sp. (capsules, pills, or tablets) | 3 | 0,1 | 3 | 0,2 | 0 | 0 |
| Bronchipret syrup (*Thymus* sp. and *Hedera helix* L.) | 3 | 0,1 | 2 | 0,1 | 1 | 0,1 |
| Umckaloabo (*Pelargonium sidoides* DC., capsules, pills, or tablets) | 2 | 0,1 | 2 | 0,1 | 0 | 0 |
| Gelodurat essential oil capsules (*Eucalyptus* sp., *Myrtus* sp., *Citrus* spp.) | 2 | 0,1 | 2 | 0,2 | 0 | 0,0 |
| Annual wormwood (*Artemisia annua* L., herbal tea) | 1 | 0 | 0 | 0 | 1 | 0,1 |
| Turnip (*Brassica rapa* L., syrup) | 1 | 0 | 0 | 0 | 1 | 0,1 |
| *Rhodiola rosea* L. (capsules, pills, or tablets) | 1 | 0 | 1 | 0,1 | 0 | 0 |
| Sinupret extract (*Genziana* sp., *Verbena* sp., *Sambucus* sp., *Rumex* sp., *Primula* sp.) | 1 | 0 | 1 | 0,1 | 0 | 0 |
| Tiger balm | 1 | 0,04 | 1 | 0,08 | 0 | 0 |
|  |  |  |  |  |  |  |
| **TCM treatments and herbal preparations** | 7 | 0,3 | 3 | 0,2 | 4 | 0,4 |
| Moxibustion | 1 | 0 | 0 | 0 | 1 | 0,1 |
| Individualized treatment | 1 | 0 | 0 | 0 | 1 | 0,1 |
| Herbal formulation (did not remember name) | 3 | 0,1 | 2 | 0,1 | 1 | 0,1 |
|  |  |  |  |  |  |  |
| **Ayurvedic preparations** | 4 | 0,2 | 4 | 0,3 | 0 | 0 |
| Amla (*Phyllanthus emblica* L.) | 1 | 0 | 1 | 0,1 | 0 | 0 |
| Anu thailam (multi compound nasal oil) | 1 | 0 | 1 | 0,1 | 0 | 0 |
| Tulsi (*Ocimum tenuiflorum* L.) | 1 | 0 | 1 | 0,1 | 0 | 0 |
|  |  |  |  |  |  |  |
| **Homeopathy** | 46 | 1,9 | 36 | 2,5 | 10 | 1 |
| Bronchi plantago | 1 | 0 | 1 | 0,1 | 0 | 0 |
| **Anthroposophy** | 3 | 0,1 | 3 | 0,2 | 0 | 0 |
| Plantago bronchial balm | 1 | 0 | 1 | 0,1 | 0 | 0 |
|  |  |  |  |  |  |  |
| **ESSENTIAL OILS (inhalation)** | 78 | 3,2 | 59 | 4,1 | 19 | 1,9 |
|  |  |  |  |  |  |  |
| **Eucalyptus (*Eucalyptus* sp.)** | 61 | 2,5 | 50 | 3,4 | 11 | 1,1 |
|  |  |  |  |  |  |  |
| **Tea Tree (*Melaleuca alternifolia***  **(Maiden & Betche) Cheel)** | 37 | 1,5 | 29 | 2 | 7 | 0,7 |
|  |  |  |  |  |  |  |
| **Vicks First Defense^®^ inhaler^®^ nasal stick^®^ (Menthol, camphor, eucalyptol)** | 24 | 1 | 20 | 1,4 | 4 | 0,4 |
|  |  |  |  |  |  |  |
| **Thyme (*Thymus* sp.)** | 21 | 0,9 | 17 | 1,2 | 4 | 0,4 |
|  |  |  |  |  |  |  |
| **Lemon (*Citrus limon* L.)** | 12 | 0,5 | 10 | 0,7 | 2 | 0,2 |
|  |  |  |  |  |  |  |
| **Olbas (*Melaleuca leucadendra* (L.) L., *Syzygium aromaticum* (L.) Merr. & L.M. Perry, *Eucalyptus* sp., *Juniperus* sp., levomenthol, methyl salicylate, *Mentha* sp.)** | 4 | 0,2 | 3 | 0,2 | 1 | 0,1 |
|  |  |  |  |  |  |  |
| **Oregano (*Origanum vulgare* L.)** | 3 | 0,1 | 1 | 0,1 | 2 | 0,2 |
|  |  |  |  |  |  |  |
| **Ravintsara (*Cinnamomum camphora***  **(L.) J. Presl)** | 3 | 0,1 | 2 | 0,1 | 1 | 0,1 |
|  |  |  |  |  |  |  |
|  |  |  |  |  |  |  |
| **DIET / FOOD** | **Total** | **%** | **Total ♀** | **%** | **Total ♂** | **%** |
| **Special foods (Dairy-free, gluten-free probiotics, honey, fruits, soups, spices)** | 107 | 4,3 | 72 | 5 | 35 | 3,5 |
| **Special diets (gluten-free, dairy-free)** | 26 | 1,1 | 17 | 1,2 | 9 | 0,9 |
| Dairy-free | 15 | 0,6 | 12 | 0,8 | 3 | 0,3 |
| Low carb diet | 9 | 0,4 | 5 | 0,3 | 4 | 0,4 |
| Gluten-free | 7 | 0,3 | 5 | 0,3 | 2 | 0,2 |
| Vegetarian | 5 | 0,2 | 2 | 0,1 | 3 | 0,3 |
| Paleo | 1 | 0 | 0 | 0 | 1 | 0,1 |
|  |  |  |  |  |  |  |
| **Fruits and vegetables** | 62 | 2,5 | 39 | 2,7 | 23 | 2,3 |
| **Soups** | 39 | 1,6 | 28 | 1,9 | 11 | 1,1 |
| **Probiotics suppl. (e.g., Yakult, Actimel)** | 23 | 0,9 | 15 | 1 | 8 | 0,8 |
| **Spices** | 23 | 0,9 | 15 | 1 | 8 | 0,8 |
| **Kombucha, kefir** | 9 | 0,4 | 7 | 0,5 | 2 | 0,2 |
| **Seaweed** | 5 | 0,2 | 3 | 0,2 | 2 | 0,2 |
|  |  |  |  |  |  |  |
| **EXERCISE OR ACTIVITIES** | 71 | 2,9 | 46 | 3,2 | 25 | 2,5 |
| Walking, hiking | 38 | 1,5 | 22 | 1,5 | 16 | 1,6 |
| Sport activities | 42 | 1,7 | 24 | 1,7 | 18 | 1,8 |
| Meditation, mindfulness | 17 | 0,7 | 12 | 0,8 | 5 | 0,5 |
| Yoga | 15 | 0,6 | 14 | 1 | 1 | 0,1 |
| Sunbathing | 10 | 0,4 | 7 | 0,5 | 3 | 0,3 |
| Massage, reflexology | 6 | 0,2 | 5 | 0,3 | 1 | 0,1 |
| Physiotherapy | 3 | 0,1 | 1 | 0,1 | 2 | 0,2 |
| Tai chi | 3 | 0,1 | 2 | 0,1 | 1 | 0,1 |
| Spa, sauna, hammam) | 1 | 0 | 0 | 0 | 1 | 0,1 |
